# Estimating Causal Effects on a Disease Progression Trait Using Bivariate Mendelian Randomisation

**DOI:** 10.1002/gepi.22600

**Published:** 2024-10-24

**Authors:** Siyang Cai, Frank Dudbridge

**Affiliations:** ^1^ Department of Population Health Sciences University of Leicester Leicester UK

**Keywords:** causality, Crohn's disease, GWAS, index event bias, instrumental variable, selection bias

## Abstract

Genome‐wide association studies (GWAS) have provided large numbers of genetic markers that can be used as instrumental variables in a Mendelian Randomisation (MR) analysis to assess the causal effect of a risk factor on an outcome. An extension of MR analysis, multivariable MR, has been proposed to handle multiple risk factors. However, adjusting or stratifying the outcome on a variable that is associated with it may induce collider bias. For an outcome that represents progression of a disease, conditioning by selecting only the cases may cause a biased MR estimation of the causal effect of the risk factor of interest on the progression outcome. Recently, we developed instrument effect regression and corrected weighted least squares (CWLS) to adjust for collider bias in observational associations. In this paper, we highlight the importance of adjusting for collider bias in MR with a risk factor of interest and disease progression as the outcome. A generalised version of the instrument effect regression and CWLS adjustment is proposed based on a multivariable MR model. We highlight the assumptions required for this approach and demonstrate its utility for bias reduction. We give an illustrative application to the effect of smoking initiation and smoking cessation on Crohn's disease prognosis, finding no evidence to support a causal effect.

## 1 Introduction

A significant benefit arising from genome‐wide association studies (GWAS) is the availability of single‐nucleotide polymorphisms (SNPs) that can serve as genetic instrumental variables (IVs) to mitigate confounding in epidemiological investigations. Associations from GWAS can be employed to deduce causal connections between phenotypes through two‐sample Mendelian randomisation (MR), which allows for the inference of causality without requiring individual‐level data (Hemani et al. 2018). While the majority of GWAS relate to the occurrence of disease, there has been a rising interest in investigating subsequent events or disease progression, which can encompass a diverse range of outcomes, including but not limited to survival, recurrence, time taken to surgery and decline in function.

The number of disease progression studies is still limited, but evidence has suggested that risk factors potentially related to disease incidence and disease progression could be of different magnitude. For example, GWAS of Crohn's disease reported distinct genetic variants associated with the risk of disease onset and disease progression, with the negative genetic correlation between the occurrence and progression of the disease (Lee et al. 2017). Similarly, a strong causal relationship between the *MUC5B* gene and the risk of idiopathic pulmonary fibrosis has been elucidated (Burgess et al. 2012), while the same gene is also associated with a much lower or even reversed effect on survival (Cai et al. 2023). Indeed it is possible that the impacts of a specific exposure on the incidence and prognosis of a disease might operate in opposite directions, and that risk factors do not always associate with progression (Mitchell et al. 2023).

It is unclear however whether such contrasting findings reflect causal effects. For instance, collider bias has been investigated in recent research as a problematic issue when conditioning on disease incidence, as must be done in studies of progression. Howe et al. (2020) discovered that case stratification distorted the associations of polygenic risk scores derived from case‐control studies, compared with populations initially free of coronary artery disease. Paternoster et al. (2017) discussed collider bias with a simulation study and provided guidelines to mitigate potential bias in disease progression research. In situations where explanatory variables can describe the conditioning process, methods such as inverse probability weighting (Monsees, Tamimi, and Kraft 2009) or likelihood approaches (Lin and Zeng 2009) can be applied to remove collider bias. When such variables are not available, IV methods related to MR are available (Cai et al. 2022). However, while these methods can adjust for collider bias in observational exposure‐progression associations, and MR can mitigate confounding of such associations, there remains a need for estimation of causal relationships that includes exposures of interest, disease incidence and disease progression and adjusts for both collider bias and confounding at the same time.

Recently, Gkatzionis et al. (2024) have addressed this problem by applying the Heckman selection model, and related techniques, to the gene‐exposure and/or gene‐outcome associations used in MR. This approach, however, requires individual level data for the trait under selection, giving a more restricted range of application than two‐sample MR. Consequently, our aim in this paper is to clarify the relationship between an exposure and disease progression, conditioning on the presence of disease, using two‐sample multivariable MR (Burgess and Thompson 2015). We demonstrate a new approach, combining IV methods to correct for both collider bias and confounding while also adjusting for weak instrument bias. We illustrate in different circumstances that this approach remains robust and adaptable.

## 2 Methods

In this section, we focus on adjusting for collider bias, confounding and weak instrument bias in a bivariate MR, with one risk factor of interest, one disease trait and a disease progression trait as an outcome. Before moving into a discussion of collider bias in disease progression, without stratification or selection on the individuals in GWAS, we can use a bivariate MR to represent the relationships in a disease progression scenario. We regard disease progression as the outcome, while we assume that there are two risk factors: the exposure whose direct effect on the outcome is our interest, and the disease incidence.

### 2.1 Multivariable MR

Burgess and Thompson (2015) developed multivariable MR to describe functional pleiotropy as a causal mechanism when genetic variants are associated with multiple risk factors. Similar assumptions to standard MR are then made for valid genetic variants in multivariable MR. We assume that for each variant:

1. The variant is associated with one or more risk factors.
2. The variant is not associated with any confounders regarding associations between risk factors and outcome.
3. The variant is independent of outcome, given risk factors and confounders.

**Figure 1:**
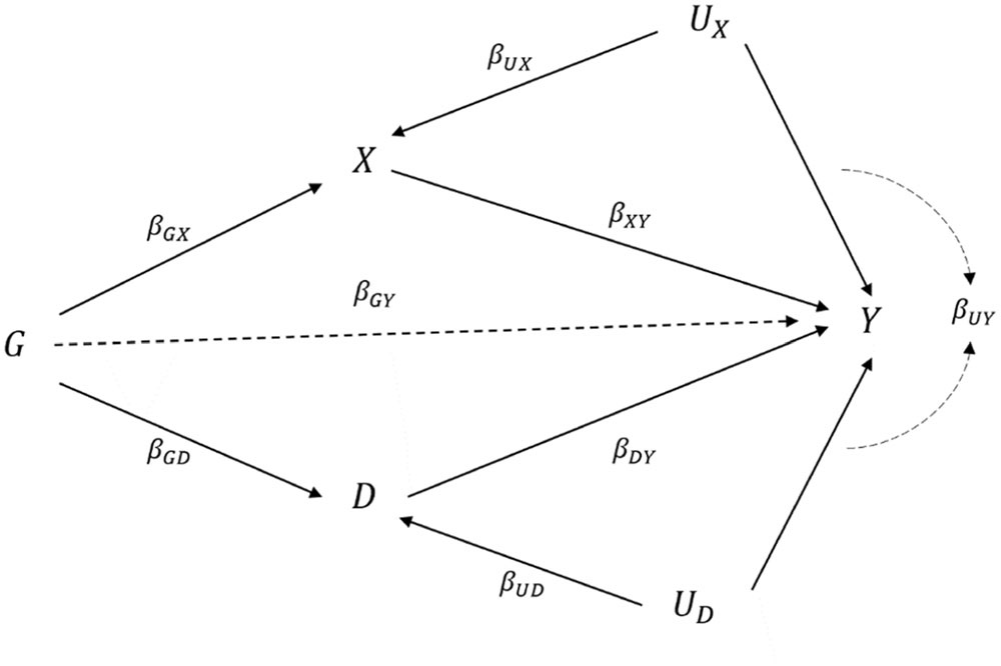
A directed acyclic graph to describe the disease progression scenario, where G is the coded genotype, $U_{X}$ and $U_{D}$ are unmeasured confounders, X is the risk factor of interest, D represents the disease trait and Y is the disease progression outcome. Notation for effect sizes is shown alongside each arrow. The total effect of confounders on Y is represented by the dotted curves. The dashed arrow from G to Y represents a potential invalid instrument having pleiotropic effect.

Multivariable MR has the capability to represent disease progression studies. Figure 1 illustrates the relationship in a directed acyclic graph, where G is the coded genotype, $U_{X}$ and $U_{D}$ are unmeasured confounders, X is the risk factor of interest, D represents the disease trait and Y is the disease progression outcome. Under the assumption of functional pleiotropy (Burgess and Thompson 2015), genetic variants are allowed to have different magnitude of effects on the exposure X and disease trait D, while it is not necessary for every genetic variant to be associated with both exposure and disease. Similarly, a genetic variant that is directly associated with the disease progression Y is referred to as “invalid” in multivariable MR.

### 2.2 Conditioning on One Risk Factor in Bivariate MR

Collider bias is a problematic issue in disease progression studies, which can be introduced by stratifying the collider or statistically adjusting for it. In Figure 1, conditioning on the risk of disease D induces a backdoor spurious path $G\to D\leftarrow U_{D}\to Y$, causing collider bias in the estimation of the effect of G on Y (Dudbridge et al. 2019; Munafò et al. 2012). If used in a standard MR analysis, this results in a biased estimation of the causal effect between the risk factor X and disease progression outcome Y (Paternoster et al. 2017). Collider bias may result in either an over‐ or under‐estimation of the causal effect, depending on the direction of relationships between the risk factors and the onset of the disease (Paternoster et al. 2017).

Consequently, in the following sections, we will introduce generalised instrument effect regression to adjust for collider bias in bivariate MR. A further discussion will be given on the impact of a causal relationship between exposure X and disease incidence D, with a combined correction approach for weak instrument bias.

### 2.3 Generalised Instrument Effect Regression

To adjust for collider bias in genetic associations, Cai et al. (2022), Dudbridge et al. (2019) and Zhu et al. (2018) developed instrument effect regression based on a linear model framework, starting from the conditional effect on the outcome and adjusting back to the direct effects of the genes. Under linear models for D and Y, the instrument effect regression is

(1) $$\beta_{\mathrm{GY}}^{C}=\beta_{\mathrm{GY}}+b\beta_{\mathrm{GD}},$$

where $\beta_{\mathrm{GY}}^{C}$ is the conditional effect of G on Y given D, $\beta_{\mathrm{GY}}$ is the direct effect of G on Y and $\beta_{\mathrm{GD}}$ is the effect of G on D. The direct genetic effects $\beta_{\mathrm{GY}}$ are the residuals of the regression of $\beta_{\mathrm{GY}}^{C}$ on $\beta_{\mathrm{GD}}$, where the regression is fitted by estimating b over a large number of SNPs.

However, in the MR context, X is another trait representing the exposure of interest, forming the bivariate MR illustrated by Figure 1. We extend the work in Dudbridge et al. (2019) to obtain a generalised version of instrument effect regression to perform MR of X while adjusting for collider bias through D.

Suppose X and D are both linear in the genotype G. We allow that the risk factors X and D do not have shared non‐genetic confounders and both have direct effects on the progression Y (Figure 1). Note that we do not assume that any SNPs are valid IVs, as in Dudbridge et al (Dudbridge et al. 2019). This forms the following equations:

$$X=\beta_{\mathrm{GX}}G+\beta_{\mathrm{UX}}U_{X}+E_{X},$$

$$D=\beta_{\mathrm{GD}}G+\beta_{\mathrm{UD}}U_{D}+E_{D},$$

$$Y=\beta_{\mathrm{GY}}G+\beta_{\mathrm{UY}}U_{Y}+\beta_{\mathrm{DY}}D+\beta_{\mathrm{XY}}X+E_{Y},$$

where $U_{X}=u_{G}+u_{x}$, $U_{D}=u_{G}+u_{d}$,$U_{Y}=u_{G}+u_{x}+u_{d}$ are combined genetic and non‐genetic common causes. The causal effect of interest is $\beta_{\mathrm{XY}}$.

Conditioning on the disease trait results in a generalised instrument effect regression for bivariate MR via the following equation:

(2) $$\beta_{\mathrm{GY}}^{C}=\beta_{\mathrm{GY}}+\beta_{\mathrm{XY}}\beta_{\mathrm{GX}}+\frac{-\beta_{\mathrm{XY}}\beta_{\mathrm{UX}}\beta_{\mathrm{UD}}\text{var}\left(u_{G}\right)-\beta_{\mathrm{UY}}\beta_{\mathrm{UD}}\text{var}\left(U_{D}\right)}{\beta_{\mathrm{UD}}^{2}{\text{var}(U}_{D})+\text{var}\left(E_{D}\right)}\beta_{\mathrm{GD}},$$

where $\beta_{\mathrm{GY}}^{C}$ is the conditional effect of G on Y given D. A derivation of Equation (2) is given in Supporting Information. This indicates that conditioning on D does not change the linear structure between the outcome and risk factors. To estimate the causal effect of the exposure of interest X on the disease progression Y, we can express the generalised instrument effect regression as a bivariate inverse variance weighted (IVW) regression model:

$$\beta_{\mathrm{GY}}^{C}\sim \beta_{\mathrm{GX}}+\beta_{\mathrm{GD}},$$

where the coefficient of $\beta_{\mathrm{GX}}$ is the causal effect $\beta_{\mathrm{XY}}$. The variables in this regression are the summary statistics observed in GWAS for exposure $\beta_{\mathrm{GX}}$, disease incidence $\beta_{\mathrm{GD}}$ and progression given disease $\beta_{\mathrm{GY}}^{C}$ and with these summary statistics the regression has the same form as multivariable MR. Specifically, the causal effect $\beta_{\mathrm{XY}}$ is the coefficient of $\beta_{\mathrm{GX}}$ in the regression of $\beta_{\mathrm{GY}}^{C}$ on $\beta_{\mathrm{GX}}$ and $\beta_{\mathrm{GD}}$, weighted by $\sigma_{GY^{C}}^{-2}$, the inverse sampling variance of the estimate ${\hat{\beta }}_{\mathrm{GY}}^{C}$.

Note that the causal effect $\beta_{\mathrm{XY}}$ also appears in the coefficient of $\beta_{\mathrm{GD}}$ in Equation (2). Thus, if the other terms in that coefficient were known, we could access more information in estimating $\beta_{\mathrm{XY}}$. However, those terms involve the unmeasured confounders and we can only identify the entire coefficient. Nevertheless, $\beta_{\mathrm{XY}}$ is identified from the coefficient of $\beta_{\mathrm{GX}}$ and we therefore use that to infer the causal effect.

### 2.4 Weak Instruments: Corrected Weighted Bivariate Least Squares (CWBLS)

As it is recommended to use a large number of SNPs to adjust for collider bias using instrument effect regression, it is generally necessary to also adjust for weak instrument bias (Cai et al. 2022; Burgess and Thompson 2011a). Weak instruments explain little variation in the exposure compared to that explained by confounders (Burgess and Thompson 2011b). Adjustment for weak instrument bias is crucial in disease progression studies, as weak instruments will tend to under‐correct for collider bias (Burgess and Thompson 2011b; Zhao et al. 2020). Cai et al. (2022) gave a formulation called *corrected weighted least squares* (CWLS) based on instrument effect regression (Equation 1). The CWLS adjustment is obtained by first reviewing the weighted least squares when the estimate of $\beta_{\mathrm{GX}}$ is disturbed by sampling error. Then the adjusted slope $b_{\mathrm{cor}}$ can be derived by comparing the observed biased slope with its true value. CWLS provides a similar adjustment for weak instrument bias compared to MR‐RAPS (Zhao et al. 2020), while MR‐RAPS is robust and more efficient in estimating the variance of the slope b in Equation (1). An identical method to CWLS, called Debiased IVW, was developed independently (Ye, Shao, and Kang 2019).

In two‐sample multivariable MR, a multivariable IVW regression is commonly used, but it is vulnerable to weak instrument bias (Burgess and Thompson 2015). Hence, we extended the CWLS estimator to a bivariate version based on weighted bivariate IVW (Supporting Information). Suppose the estimated effects ${\hat{\beta }}_{G_{j}X},{\hat{\beta }}_{G_{j}D}$ and ${\hat{\beta }}_{G_{j}Y}^{C}$ are the summary statistics for SNP j corresponding to exposure, disease and disease progression conditioning on disease, with $\sigma_{G_{j}X}^{2}$, $\sigma_{G_{j}D}^{2}$ and $\sigma_{G_{j}Y}^{2}$ the squared standard errors assumed known. Then a corrected estimator of the coefficient of $\beta_{\mathrm{XY}}$ in the bivariate IVW regression of Equation (2) can be derived as

$${\hat{b}}_{\mathrm{CWBLS}}=\frac{\sum w_{j}{\hat{\beta }}_{G_{j}D}^{2}\sum w_{j}{\hat{\beta }}_{G_{j}X}\;{\hat{\beta }}_{G_{j}Y}^{C}-\sum w_{j}{\hat{\beta }}_{G_{j}X}{\hat{\beta }}_{G_{j}D}\sum w_{j}{\hat{\beta }}_{G_{j}D}{\hat{\beta }}_{G_{j}Y}^{C}-N}{\sum w_{j}{\hat{\beta }}_{G_{j}X}^{2}\sum w_{j}{\hat{\beta }}_{G_{j}D}^{2}-{\left(\sum w_{j}{\hat{\beta }}_{G_{j}X}{\hat{\beta }}_{G_{j}D}\right)}^{2}-D},$$

where the sums are over j and

$$N\approx \sum w_{j}\sigma_{G_{j}D}^{2}\sum w_{j}{\hat{\beta }}_{G_{j}X}{\hat{\beta }}_{G_{j}Y}^{C},$$

$$D\approx \sum w_{j}\sigma_{G_{j}D}^{2}\sum w_{j}{\hat{\beta }}_{G_{j}X}^{2}+\sum w_{j}\sigma_{G_{j}X}^{2}\sum w_{j}{\hat{\beta }}_{G_{j}D}^{2}-\sum w_{j}\sigma_{G_{j}X}^{2}\sum w_{j}\sigma_{G_{j}D}^{2},$$

$$w_{j}=\sigma_{G_{j}Y}^{-2}.$$

We call this approach CWBLS. To evaluate the standard error of this estimator, we recommend non‐parametric bootstrapping by repeatedly resampling the summary statistics from their estimated sampling distributions.

### 2.5 Causal Relationship Between Exposure and Disease

**Figure 2:**
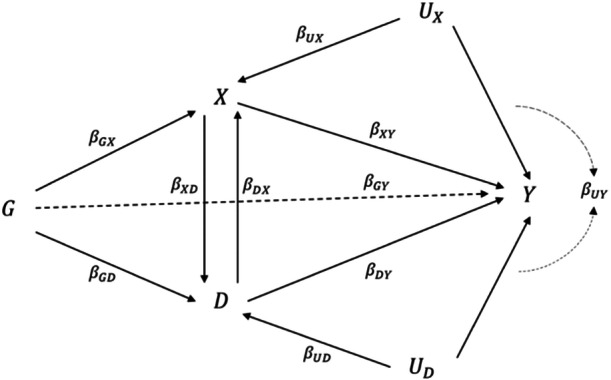
A directed acyclic graph to describe the disease progression scenario, where G is the coded genotype, $U_{X}$ and $U_{D}$ are unmeasured confounders, X is the risk factor of interest, D represents the disease trait and Y is the disease progression outcome. The assumption of causal independence can be violated with a non‐zero causal effect between X and D. The arrows between X and D represent a single effect that could be in either direction, not bidirectional effects. The dashed arrow from G to Y represents a potential invalid instrument having pleiotropic effect.

The assumption of causal independence of exposure and disease traits can be relaxed by allowing causal effects between them, which may be a more plausible scenario in applications. For instance, decreasing the level of low‐density lipoprotein cholesterol (LDL‐C) is causally protective against the risk of coronary heart disease (CHD) (Ference et al. 2013), while the causal effect of LDL‐C on progression from CHD such as a cardiovascular or fatal outcome is of interest for the treatment of disease (Howe et al. 2020). In the notation of Figure 2, LDL‐C plays the role of exposure X, while disease trait D is CHD and disease progression Y is the subsequent event of CHD.

Paternoster et al. (2017). stated that the direction of such a causal relationship also has an impact on the direction of collider bias. To investigate the impact of a causal relationship between the exposure of interest and disease trait on the instrument effect regression, we either replace the exposure X with:

$$X=\beta_{\mathrm{GX}}G+\beta_{\mathrm{UX}}U_{X}+\beta_{\mathrm{DX}}D+E_{X},$$

or the disease trait D with:

$$D=\beta_{\mathrm{GD}}G+\beta_{\mathrm{UD}}U_{D}+\beta_{\mathrm{XD}}X+E_{D},$$

depending on the assumed direction of the causal relationship. In the first case, where the disease trait D has causal effect on exposure X, this results in the same generalised instrument effect regression as before (Equation 2):

$$\beta_{\mathrm{GY}}^{C}=\beta_{\mathrm{GY}}+\beta_{\mathrm{XY}}\beta_{\mathrm{GX}}+\frac{-\beta_{\mathrm{XY}}\beta_{\mathrm{UX}}\beta_{\mathrm{UD}}\text{var}\left(u_{G}\right)-\beta_{\mathrm{UY}}\beta_{\mathrm{UD}}\text{var}\left(U_{D}\right)}{\beta_{\mathrm{UD}}^{2}{\text{var}(U}_{D})+\text{var}\left(E_{D}\right)}\beta_{\mathrm{GD}}.$$

This is because the multivariable MR implicitly conditions on D, blocking the path from D to X. However in practice, GWAS of X may estimate the marginal effect of G, that is $\beta_{\mathrm{GX}}^{M}=\beta_{\mathrm{GX}}+\beta_{\mathrm{GD}}\beta_{\mathrm{DX}}$ rather than the direct effect $\beta_{\mathrm{GX}}$. Note also that the marginal effect on D is just $\beta_{\mathrm{GD}}^{M}=\beta_{\mathrm{GD}}$. Substituting into Equation (2) we have:

(3) $$\beta_{\mathrm{GY}}^{C}=\beta_{\mathrm{GY}}+\beta_{\mathrm{XY}}\beta_{\mathrm{GX}}^{M}+\left(\frac{-\beta_{\mathrm{XY}}\beta_{\mathrm{UX}}\beta_{\mathrm{UD}}\text{var}\left(u_{G}\right)-\beta_{\mathrm{UY}}\beta_{\mathrm{UD}}\text{var}\left(U_{D}\right)}{\beta_{\mathrm{UD}}^{2}{\text{var}(U}_{D})+\text{var}\left(E_{D}\right)}-\beta_{\mathrm{XY}}\beta_{\mathrm{DX}}\right)\beta_{\mathrm{GD}}^{M}.$$

In both Equations (2) and (3), the causal effect is the coefficient of the effect of G on X, whether marginal or conditional on D.

On the other hand, if exposure X has a causal effect on the disease trait D, the generalised instrument effect regression becomes

$$\beta_{\mathrm{GY}}^{C}=\beta_{\mathrm{GY}}+\beta_{\mathrm{XY}}\beta_{\mathrm{GX}}+\frac{\mathrm{numer}}{\mathrm{denom}}\left(\beta_{\mathrm{GD}}+\beta_{\mathrm{GX}}\beta_{\mathrm{XD}}\right),$$

where

$$\mathrm{numer}=-\;\left[\beta_{\mathrm{XY}}\beta_{\mathrm{XD}}\beta_{\mathrm{UX}}^{2}\text{var}\left(U_{X}\right)+\beta_{\mathrm{UY}}\beta_{\mathrm{UD}}\text{var}\left(U_{D}\right)+\beta_{\mathrm{UY}}\beta_{\mathrm{XD}}\beta_{\mathrm{UX}}\text{var}\left(U_{X}\right)+\beta_{\mathrm{XY}}\beta_{\mathrm{UX}}\beta_{\mathrm{UD}}\text{var}\left(u_{G}\right)\right],$$

and

$$\mathrm{denom}=\;\beta_{\mathrm{UD}}^{2}\text{var}\left(U_{D}\right)+\beta_{\mathrm{XD}}^{2}\beta_{\mathrm{UX}}^{2}\text{var}\left(U_{X}\right)+2\beta_{\mathrm{XD}}\beta_{\mathrm{UD}}\beta_{\mathrm{UX}}\text{var}\left(u_{G}\right)+\text{var}\left(E_{D}\right)+\beta_{\mathrm{XD}}^{2}\text{var}\left(E_{X}\right).$$

A derivation is given in Supporting Information. If, as is often the case, GWAS of D provide the marginal effects $\beta_{\mathrm{GD}}^{M}=\beta_{\mathrm{GD}}+\beta_{\mathrm{GX}}\beta_{\mathrm{XD}}$, substituting for $\beta_{\mathrm{GD}}$ yields

(4) $$\beta_{\mathrm{GY}}^{C}=\beta_{\mathrm{GY}}+\beta_{\mathrm{XY}}\beta_{\mathrm{GX}}^{M}+\frac{\mathrm{numer}}{\mathrm{denom}}\;\beta_{\mathrm{GD}}^{M}.\;$$

Equations (3) and (4) imply that violating the assumption of causal independence does not change the overall structure of generalised instrument effect regression, provided that the marginal associations of G are used. Hence, CWBLS remains capable of adjusting for weak instrument bias based on bivariate IVW to correct for collider bias under the existence of causal dependence between two risk factors.

### 2.6 Summary of Methods

We have argued that for two‐sample MR with a disease progression outcome, the exposure and disease risk can be considered in a bivariate analysis in which the causal effect of the exposure is obtained from a multivariable IVW analysis using the observed (i.e., conditional on disease) genetic effects on the outcome. The standard assumptions of multivariable MR apply, with the additional assumptions described below. Furthermore, as disease is a binary trait we assume that the effects $\beta_{\mathrm{GD}}$ are sufficiently small for linear models to approximately hold (Dudbridge et al. 2019). In practice, weak instruments may be present, for which we propose CWBLS.

To summarise the additional assumptions that are required in our approach:

1. Measurement errors in estimated marginal effects of exposure and disease traits are independent. This is plausible under a two‐ or three‐sample MR, in which the effects on exposure are estimated in one sample, and those on the disease and progression are estimated in another sample or separately.
2. An instrument coefficient linearly uncorrelated with direct effect (InCLUDE) assumption is required, similar to (Cai et al. 2022) and analogous to the InSIDE assumption of MR (Bowden, Davey Smith, and Burgess 2015; Sanderson et al. 2019; Grant and Burgess 2021). This requires the direct effects $\beta_{\mathrm{GY}}$ to be uncorrelated with genetic effects $\beta_{\mathrm{GX}}$ and $\beta_{\mathrm{GD}}$. However, the InCLUDE assumption can be disrupted in disease risk GWAS which commonly assume that associated SNPs provide targets for treatment (Cai et al. 2022), or residual correlation due to random variation (Grant and Burgess 2021). This provides the motivation of using as many genetically independent instruments as possible to perform the adjustment so that the correlation among the genetic effects may tend to zero. This can be performed using a set of pruned variants with high imputation quality, or other methods such as principal components analysis (PCA) to perform dimension reduction on the set of genetic variants used.
3. In common with multivariate MR, we assume no interactions in the effects of G on X, D and Y. This is especially pertinent to the effect of G on the exposure X as it implies that the direct effect $\beta_{\mathrm{GX}}$, which appears in the marginal effect $\beta_{\mathrm{GX}}^{M}$ used in our approach, is the same in the cases and non‐cases of disease D. It may be that genetic effects on an exposure change in the presence of a disease, in which case MR estimation of $\beta_{\mathrm{XY}}$ would require GWAS of X among cases only.

There are several other methods available to correct for weak instrument bias in multivariate MR. MR‐GRAPPLE (Wang et al. 2021) is the multivariate generalisation of MR‐RAPS using a profile score approach. Batool et al. (2022) derived a multivariable version of limited information maximum likelihood (MV‐LIML) which remains robust when using weak instruments. Debiased IVW (Wu, Kang, and Ye 2024) is also a two‐sample multivariate MR method that corrects for weak instrument bias based on an asymptotic analysis of the MV‐IVW estimator. While the univariate Debiased IVW is identical to CWLS, the multivariate version differs from our CWBLS. Furthermore, MRBEE (Lorincz‐Comi et al. 2024) is capable of simultaneously removing weak instrument and sample overlap bias and identifying horizontal pleiotropy using a score‐based estimator and bias‐corrected estimating equation. We will apply MR‐GRAPPLE, MV‐LIML, Debiased IVW and MRBEE in the next section to compare results with CWBLS in different simulation scenarios.

### 2.7 Simulations

To evaluate the ability of CWBLS to estimate the causal effect on disease progression shown in Figure 1, we carried out similar simulations to previous studies (Cai et al. 2022; Burgess and Thompson 2015; Dudbridge et al. 2019). We generate data for n=20,000 individuals indexed by i, and m=10,000 independent SNPs indexed by j on one exposure X, one disease D and a disease progression outcome Y from the following data‐generating model:

$$x_{i}=g_{x_{i}}+\beta_{\mathrm{XD}}d_{i}+u_{x_{i}}+\epsilon_{x_{i}},$$

$$d_{i}=g_{d_{i}}+\beta_{\mathrm{DX}}x_{i}+u_{d_{i}}+\epsilon_{d_{i}},$$

$$y_{i}=g_{y_{i}}+\beta_{\mathrm{XY}}x_{i}+\beta_{\mathrm{DY}}d_{i}+u_{x_{i}}+u_{d_{i}}+\epsilon_{y_{i}},$$

$$u_{x_{i}}\sim N\left(0,\sigma_{u_{x}}^{2}\right),u_{d_{i}}\;\sim N\left(0,\sigma_{u_{d}}^{2}\right),$$

where $g_{x_{i}}$ is the sum of genetic effects $\sum_{j=1}^{m}\beta_{G_{j}X}G_{\mathrm{ij}}$ normalised to follow $N(0,h_{\mathrm{GX}}^{2})$, $g_{d_{i}}$ is the sum of genetic effects $\sum_{j=1}^{m}\beta_{G_{j}D}G_{\mathrm{ij}}$ normalised to follow $N\left(0,h_{\mathrm{GD}}^{2}\right)$ and $g_{y_{i}}$ is the sum of genetic effects $\sum_{j=1}^{m}\beta_{G_{j}Y}G_{\mathrm{ij}}$ normalised to follow $N\left(0,h_{\mathrm{GY}}^{2}\right)$. Initially, a total fixed number of m=10,000 SNPs was simulated under Hardy‐Weinberg equilibrium with minor allele frequencies drawn uniformly from (0.01,0.49). The SNP effects $\beta_{G_{j}X}$, $\beta_{G_{j}D}$ and $\beta_{G_{j}Y}$ are independently simulated from N(0,1) so that weak instrument bias emerges when the total number of instruments is large enough. $h_{\mathrm{GX}}^{2}$ and $h_{\mathrm{GD}}^{2}$ are the heritabilities which are varied to take values of 0.3 or 0.1. The pleiotropic variance $h_{\mathrm{GY}}^{2}$ is set to 0 to produce valid instruments that have independent effects on both X and D but not directly on the outcome Y. When simulating invalid instruments, $h_{\mathrm{GY}}^{2}$ is also varied to take the value of 0.3 or 0.1. Note that in this case, we regard all SNPs as invalid instruments. Causal dependency is explained by $\beta_{\mathrm{XD}}$ or $\beta_{\mathrm{DX}}$ with respect to the direction, choosing values from ±0.2 or ±0.5 and the other as 0. The true causal effect that we are interested in is $\beta_{\mathrm{XY}}$, with the direct effect $\beta_{\mathrm{DY}}$ generally fixed at 0.

Non‐genetic confounders $u_{x_{i}}$ and$\;u_{d_{i}}$ are then simulated independently from normal distributions, where $\sigma_{u_{x}}^{2}$ and $\sigma_{u_{d}}^{2}$ are fixed at 0.2 throughout. The residuals in each trait follow independent normal distributions, such that each trait in the additive model results in a standard normal distribution.

To better describe the diagnosis of disease in a more realistic case, we simulated a second scenario treating disease D as a binary event, by assuming the liability threshold model such that 20% of individuals were affected by the disease. We then simulated a total population of n=54,000 individuals so that a sample of 10,000 cases and 10,000 controls was always available across the simulations.

To estimate SNP‐exposure, SNP‐disease and SNP‐progression beta coefficients, we simulated one sample of genetic data to obtain SNP‐exposure estimates by linear regression of exposure X on genotype. We then simulated a second sample from which SNP‐disease effects were obtained from a linear regression of disease D on genotype, as well as SNP‐progression effects from a linear regression of disease progression Y on genotype, conditioning on D as a covariate. In the scenario where disease D is binary, a logistic regression is used instead to obtain estimated SNP‐disease effects. Previous work (Cai et al. 2022) has established that for small effects, this approach is theoretically and practically similar to a case‐only analysis of a binary disease trait. The two samples are both simulated for 20,000 individuals. Estimated causal effects ${\hat{\beta }}_{\mathrm{XY}}$ from 1000 simulations are then obtained using multivariable IVW, CWBLS, MR‐GRAPPLE, MV‐LIML, Debiased IVW and MRBEE. Coverage rates for the estimated causal effect ${\hat{\beta }}_{\mathrm{XY}}$ are calculated as the probability of including the true value of $\beta_{\mathrm{XY}}$ within the estimated 95% confidence intervals. This is different from (Burgess and Thompson 2015; Slob and Burgess 2020) where the interest is in whether a method is able to detect a causal effect. Means of robust standard errors and the empirical standard deviations of the estimates are also obtained.

As we assume no linkage disequilibrium between variants, the Ω(θ) matrix in MV‐LIML (Batool et al. 2022) can be simplified to an identity matrix, greatly boosting the computational efficiency of MV‐LIML. Similarly, we may use the identity matrix as the genetic correlation matrix in MR‐GRAPPLE (default settings, Tukey loss function), Debiased IVW (default settings) and MRBEE (default settings). As the true genetic effects are simulated independently, all instruments are used in the above methods without P value selection to reduce the total number.

## 3 Results

Table 1 shows the mean estimates with corresponding coverage, mean of standard errors and standard deviation of the estimates for multivariable IVW, CWBLS and MR‐GRAPPLE, under different combinations of heritability in exposure X and disease D. The unadjusted multivariable IVW is only unbiased when there is no causal effect, otherwise showing a bias towards zero, while CWBLS and MR‐GRAPPLE perform adjustment toward true causal effects in all circumstances with significantly improved coverage rates. The results of all methods for weak instrument adjustment are very similar (Supporting Information S1: Table 1).

**Table 1:** Results from the simulation study of multivariable IVW (MVIVW), CWBLS and MR‐GRAPPLE to estimate the causal effect on disease progression, without causal relationship between exposure of interest and disease liability. Disease trait is simulated as a continuous trait.

$\left(h_{\mathrm{GX}}^{2},h_{\mathrm{GD}}^{2}\right)$	$\beta_{\mathrm{XY}}$	MVIVW				CWBLS				MR‐GRAPPLE			
		EST	CVG	SE	SD	EST	CVG	SE	SD	EST	CVG	SE	SD
(0.3, 0.3)	0.4	0.1501	56.2	0.0090	0.0269	0.4002	94.9	0.0253	0.0244	0.3960	96.4	0.0249	0.0236
(0.3, 0.1)		0.1501	55.8	0.0091	0.0254	0.3998	95.3	0.0254	0.0244	0.3955	95.9	0.0250	0.0228
(0.1, 0.3)		0.0669	27.4	0.0105	0.0586	0.4024	96.2	0.0684	0.0651	0.3996	96.3	0.0647	0.0579
(0.1, 0.1)		0.0669	27.8	0.0105	0.0587	0.4031	96.3	0.0691	0.0654	0.3998	96.4	0.065	0.0580
(0.3, 0.3)	0	0.0001	54.0	0.0079	0.0209	0.0008	96.1	0.0212	0.0209	0.0007	95.9	0.0210	0.0207
(0.3, 0.1)		0.0001	55.0	0.0078	0.0290	0.0008	96.0	0.0213	0.0215	0.0008	96.0	0.0210	0.0209
(0.1, 0.3)		0.0001	25.9	0.0091	0.0183	0.0022	95.9	0.0562	0.0540	0.0021	95.4	0.0540	0.051
(0.1, 0.1)		0.0001	26.5	0.0092	0.055	0.0023	96.3	0.0568	0.0552	0.0021	95.5	0.0542	0.0534
(0.3, 0.3)	−0.4	−0.1500	56.4	0.0078	0.0236	−0.4015	95.3	0.0222	0.0220	−0.3973	96.5	0.0220	0.0215
(0.3, 0.1)		−0.1499	55.8	0.0079	0.0197	−0.4007	95.6	0.0224	0.0217	−0.3963	96.3	0.0220	0.0200
(0.1, 0.3)		−0.0666	27.9	0.0092	0.0499	−0.4064	95.6	0.0602	0.0587	−0.3978	96.2	0.0571	0.0510
(0.1, 0.1)		−0.0666	28.2	0.0092	0.0499	−0.4071	95.9	0.0608	0.0588	−0.3992	96.2	0.0573	0.0510

Abbreviations: CVG, coverage of 95% confidence interval; EST, mean estimate over 1000 simulations; SD, empirical standard deviation of estimates; SE, mean of estimated standard errors; $h_{GX}^{2}$, $h_{GD}^{2}$, heritabilities of the exposure and disease trait; $\beta_{XY}$, true causal effect between the exposure and disease progression.

Although the mean point estimates are similar, coverage rates from using MV‐LIML are significantly lower than those generated by the other methods. This is due to the fact that MV‐LIML produces causal estimates with lower standard errors than the true standard deviations of its estimates (Batool et al. 2022). This results in unbiased estimation in the point estimate, but under‐estimated standard error and therefore lower coverage rates over the simulation samples.

Table 2 and Supporting Information S1: Table 2 display the same scenario with disease D simulated as a binary event. We observe a similar pattern to Table 1, in line with previous work (Cai et al. 2022; Dudbridge et al. 2019). Results from Tables 1 and 2 not only show that the CWBLS has good performance in disease progression scenarios compared to other proven methods where disease incidence is a binary trait, but also generalise the use of CWBLS to analyses conditioning on a continuous risk factor.

**Table 2:** Results from the simulation study of multivariable IVW (MVIVW), CWBLS and MR‐GRAPPLE to estimate the causal effect on disease progression. Details as in Table 1, with disease simulated as a binary trait with 20% prevalence.

$\left(h_{GX}^{2},h_{GD}^{2}\right)$	$\beta_{XY}$	MVIVW				CWBLS				MR‐GRAPPLE			
		EST	CVG	SE	SD	EST	CVG	SE	SD	EST	CVG	SE	SD
(0.3, 0.3)	0.4	0.1497	56.2	0.0091	0.0239	0.4007	95.1	0.0251	0.0254	0.3966	95.7	0.0249	0.0241
(0.3, 0.1)		0.1497	55.8	0.0091	0.0233	0.4005	95.6	0.0254	0.0249	0.3961	96.0	0.0250	0.0236
(0.1, 0.3)		0.0665	27.4	0.0105	0.0583	0.4033	97.0	0.0678	0.0645	0.3982	97.0	0.0644	0.0582
(0.1, 0.1)		0.0664	27.8	0.0105	0.0582	0.4037	96.5	0.0688	0.0650	0.3990	96.5	0.0647	0.0584
(0.3, 0.3)	0	−0.0004	54.0	0.0079	0.0208	0.0001	95.0	0.0210	0.0208	0.0001	94.8	0.0209	0.0206
(0.3, 0.1)		−0.0004	55.0	0.0079	0.0210	0.0001	94.9	0.0212	0.0210	0.0001	95.2	0.0210	0.0208
(0.1, 0.3)		−0.0003	25.9	0.0091	0.0544	0.0010	95.5	0.0557	0.0548	0.0010	95.6	0.0537	0.0530
(0.1, 0.1)		−0.0003	26.5	0.0091	0.0553	0.0006	95.4	0.0564	0.0559	0.0006	94.6	0.0540	0.0539
(0.3, 0.3)	−0.4	−0.1504	56.4	0.0079	0.0200	−0.4004	94.0	0.0221	0.0222	−0.3963	96.3	0.0219	0.0204
(0.3, 0.1)		−0.1504	55.8	0.0079	0.0199	−0.4008	94.3	0.0223	0.0222	−0.3964	96.2	0.0220	0.0202
(0.1, 0.3)		−0.0671	27.9	0.0091	0.0515	−0.4018	95.7	0.0598	0.0595	−0.3988	96.1	0.0568	0.0519
(0.1, 0.1)		−0.0671	28.2	0.0091	0.0518	−0.4047	95.0	0.0606	0.0608	−0.3974	95.8	0.0572	0.0526

It is also critical to investigate the performance of the methods when there is a causal relationship between the exposure X and the disease liability D. If the marginal SNP effects on X and D are directly simulated as two independent total effects, the results are exactly the same as those presented in Table 1, regardless of strength and direction of effect between X and D. However, if we simulate independent direct effects $\beta_{\mathrm{GX}}$ and $\beta_{\mathrm{GD}}$ then an effect between X and D will cause the marginal effects $\beta_{\mathrm{GX}}^{M}$ and $\beta_{\mathrm{GD}}^{M}$ to become correlated.

In Table 3 and Supporting Information S1: Table 3, we give results using genetically correlated X and D traits by taking different causal strengths and directions between X and D, with disease D as a continuous trait. The causal $\beta_{\mathrm{XY}}$ is fixed at 0.4. While multivariable IVW remains biased with zero coverage rates under all scenarios, the point estimates from CWBLS are not biased under various causal effects between X and D. CWBLS obtains similar causal estimates and coverages compared to MR‐GRAPPLE, Debiased IVW and MRBEE. However, we still observe under‐estimated standard errors using MV‐LIML, causing low coverage rates.

**Table 3:** Results from the simulation study of multivariable IVW (MVIVW), CWBLS and MR‐GRAPPLE to estimate the causal effect on disease progression $\beta_{\mathrm{XY}}=0.4$, with a causal relationship between exposure of interest and disease liability. Heritabilities of the exposure and disease trait are both fixed at 0.3. A disease trait is simulated as a continuous trait.

Effect between D and X	Direction	MVIVW				CWBLS				MR‐GRAPPLE			
		EST	CVG	SE	SD	EST	CVG	SE	SD	EST	CVG	SE	SD
0.2	X to D	0.1339	0	0.0082	0.0084	0.4007	94.7	0.0266	0.0265	0.3955	96.0	0.0263	0.0243
0.5		0.0863	0	0.0082	0.0081	0.4021	95.1	0.0334	0.0335	0.3975	96.2	0.0326	0.0291
−0.2		0.1527	0	0.0085	0.0086	0.4000	94.6	0.0261	0.0259	0.3957	95.7	0.0259	0.0242
−0.5		0.1312	0	0.0086	0.0087	0.4005	95.0	0.0321	0.0320	0.3998	95.4	0.0315	0.0292
0.2	D to X	0.1394	0	0.0083	0.0082	0.4004	95.6	0.0262	0.0248	0.3977	96.8	0.0257	0.0228
0.5		0.1196	0	0.0080	0.0078	0.4009	95.9	0.0289	0.0271	0.3987	96.9	0.0283	0.0243
−0.2		0.1581	0	0.0083	0.0084	0.4001	94.6	0.0253	0.0248	0.3991	95.7	0.0248	0.0232
−0.5		0.1641	0	0.0080	0.0082	0.4002	94.7	0.0264	0.0265	0.3995	95.2	0.0259	0.0248

Abbreviations: CVG, coverage of 95% confidence interval; EST, mean estimate over 1000 simulations; SD, empirical standard deviation of estimates; SE, mean of estimated standard errors.

Finally, we investigate the impact from using invalid instruments in CWBLS compared to other bias‐correction methods (Zhao et al. 2020) Based on the previous simulation, all SNPs are simulated as invalid instruments with balanced pleiotropy. The heritabilities of each trait are similarly chosen from 0.1 to 0.3 to create 8 different cases to estimate $\beta_{\mathrm{XY}}$ taking values −0.4, 0 and 0.4. For simplicity, we assume that ${\beta_{\mathrm{DX}}\;=\beta }_{\mathrm{DY}}\;=\;0$ to avoid repetitive work.

Results for this scenario are given in Table 4 and Supporting Information: Table 4. Multivariable IVW is unbiased only under no causal effect, with low coverage rates. Using CWBLS, Debiased IVW and MRBEE provide unbiased mean estimates under all circumstances, with improved coverage rates compared to multivariable IVW. However, causal estimates using MV‐LIML are biased in all simulations with different heritabilities when the causal effect is not zero. Low coverage rates and biased estimates indicate that MV‐LIML performs worse than CWBLS when using invalid instruments. The magnitude of bias in the causal estimates using MV‐LIML is related to the pleiotropic variance of the disease progression trait Y when the heritabilities of X and D are fixed. Moreover, we were surprised to observe a slight deviation from the nominal level in the estimates and coverage rates from MR‐GRAPPLE. This deviation seems to depend on the size of pleiotropic variance relative to the exposure heritability. This may be a vulnerability when including a large number of instruments without P‐value selection, potentially including SNPs that bring in a large pleiotropic variance.

**Table 4:** Results from the simulation study of multivariable IVW (MVIVW), CWBLS and MR‐GRAPPLE with all invalid genetic instruments. Disease trait is simulated as a continuous trait.

$\left(h_{GX}^{2},h_{GD}^{2},h_{GY}^{2}\right)$	$\beta_{XY}$	MVIVW				CWBLS				MR‐GRAPPLE			
		EST	CVG	SE	SD	EST	CVG	SE	SD	EST	CVG	SE	SD
(0.3, 0.3, 0.3)	0.4	0.1495	0	0.0084	0.0102	0.4003	95.4	0.0301	0.0290	0.4116	93.1	0.0299	0.0305
	0	−0.0004	89.8	0.0078	0.0096	−0.0007	94.7	0.0268	0.0261	−0.0018	95.1	0.0285	0.0280
	−0.4	−0.1503	0	0.0071	0.0073	−0.4004	94.0	0.0221	0.0222	−0.3963	96.3	0.0219	0.0204
(0.3, 0.3, 0.1)	0.4	0.1496	0	0.0084	0.0092	0.4007	94.7	0.0269	0.0268	0.4020	95.2	0.0261	0.0276
	0	−0.0003	93.4	0.0078	0.0086	−0.0004	94.3	0.0232	0.0234	−0.0006	94.4	0.0234	0.0237
	−0.4	−0.1502	0	0.0071	0.0080	−0.4013	94.9	0.0242	0.0241	−0.4048	93.0	0.0230	0.0245
(0.3, 0.1, 0.3)	0.4	0.1495	0	0.0084	0.0101	0.4005	95.5	0.0302	0.0291	0.4116	93.4	0.0301	0.0306
	0	−0.0004	90.4	0.0078	0.0096	−0.0008	95.1	0.0270	0.0261	−0.0018	95.0	0.0288	0.0282
	−0.4	−0.1503	0	0.0071	0.0088	−0.4021	96.5	0.0278	0.0264	−0.4225	87.5	0.0277	0.0277
(0.3, 0.1, 0.1)	0.4	0.1496	0	0.0084	0.0092	0.4009	94.8	0.0271	0.0269	0.4021	93.3	0.0262	0.0278
	0	−0.0003	93.5	0.0077	0.0085	−0.0002	94.7	0.0233	0.0234	−0.0004	95.0	0.0235	0.0237
	−0.4	−0.1502	0	0.0071	0.0078	−0.4008	95.7	0.0242	0.0236	−0.4045	93.6	0.0231	0.0241
(0.1, 0.3, 0.3)	0.4	0.0663	0	0.0097	0.0122	0.4030	94.8	0.0807	0.0802	0.4396	90.3	0.0808	0.0872
	0	−0.0003	88.2	0.0090	0.0115	−0.0020	94.4	0.0712	0.0713	−0.0047	94.6	0.0859	0.0873
	−0.4	−0.0669	0	0.0082	0.0110	−0.4066	95.9	0.0745	0.0748	−0.4701	83.9	0.0748	0.0806
(0.1, 0.3, 0.1)	0.4	0.0664	0	0.0097	0.0107	0.4037	94.9	0.0725	0.0722	0.4081	92.4	0.0680	0.0742
	0	−0.0002	93.0	0.0090	0.0100	−0.0012	94.8	0.0617	0.0620	−0.0017	94.4	0.0627	0.0638
	−0.4	−0.0668	0	0.0082	0.0093	−0.4062	96.0	0.0652	0.0641	−0.4157	92.6	0.0598	0.0651
(0.1, 0.1, 0.3)	0.4	0.0663	0	0.0097	0.0121	0.4036	95.1	0.0814	0.0806	0.4398	90.3	0.0813	0.0878
	0	−0.0003	87.9	0.0090	0.0115	−0.0020	94.5	0.0719	0.0715	−0.0046	94.2	0.0864	0.0875
	−0.4	−0.0669	0	0.0082	0.0109	−0.4074	96.1	0.0753	0.0751	−0.4693	84.2	0.0754	0.0811
(0.1, 0.1, 0.1)	0.4	0.0664	0	0.0097	0.0107	0.4043	95.2	0.0732	0.0726	0.4087	92.2	0.0684	0.0748
	0	−0.0002	93.3	0.0089	0.0099	−0.0012	94.8	0.0622	0.0623	−0.0018	94.5	0.0631	0.0641
	−0.4	−0.0668	0	0.0082	0.0094	−0.4059	95.3	0.0657	0.0662	−0.4161	90.8	0.0603	0.0681

Abbreviations: CVG, coverage of 95% confidence interval; EST, mean estimate over 1000 simulations; SD, empirical standard deviation of estimates; SE, mean of estimated standard errors; $h_{GX}^{2}$, $h_{GD}^{2}$, heritabilities of the exposure and disease trait; $h_{GY}^{2}$, variance of direct pleiotropic effects on disease progression; $\beta_{XY}$, true causal effect between the exposure and disease progression.

### 3.1 Crohn's Disease

Crohn's disease is one of the main forms of inflammatory bowel disease (IBD), affecting more than 2.5 million people in Europe (Liu et al. 2015), with increasing prevalence in other countries. GWAS of Crohn's disease susceptibility has been published by the International Inflammatory Bowel Disease Genetics Consortium (Liu et al. 2015). Lee et al. (2017) performed a subsequent study of Crohn's disease prognosis, defined as a binary phenotype consisting of good prognosis (indolent disease that requires no surgery or immunomodulators after a minimum of 4‐year follow‐up) and poor prognosis (frequently flaring, treatment‐refractory disease that requires at least 2 immunomodulators, or at least 2 surgeries, or both). Smoking behaviour has been shown to be a critical risk factor for developing Crohn's disease but the association with progression is unclear, varying from positive, negative or no relationship in different studies, due to a lack of uniformity in the definition of smoking status (Parkes, Whelan, and Lindsay 2014). For instance, Aldhous et al. (2007) demonstrated that smoking habit was associated with the diagnosis of Crohn's disease, but had little effect on progression to various severe outcomes such as perianal penetrating disease, or time taken to surgery.

To investigate the causal relationship between smoking behaviour and Crohn's disease progression, we utilised GWAS for smoking initiation and smoking cessation as two different exposures of interest (Liu et al. 2019), including up to 1.2 million individuals. Total numbers of 11,802,365 and 12,197,133 SNPs are recorded in the two GWAS summary statistics datasets respectively. We also used summary statistics for Crohn's disease incidence (5956 cases and 14,927 controls) (Liu et al. 2015) and prognosis (2734 cases) (Lee et al. 2017) that include 7,908,787 SNPs, which were combined and analysed for index event bias in (Dudbridge et al. 2019). To estimate the causal effects, we used a pruned set ($r^{2}$ ≤ 0.1, 250 SNP window) of 131,121 well‐imputed SNPs ($R^{2}\geq 0.99$). This forms a two‐sample bivariate MR, with smoking initiation or smoking cessation as our exposure of interest, Crohn's disease incidence as a disease trait and Crohn's disease prognosis as disease progression trait. The directed acyclic graph is as Figure 2, as smoking initiation and cessation both have potential direct effects on Crohn's disease susceptibility (Parkes, Whelan, and Lindsay 2014; Aldhous et al. 2007; Song et al. 2018).

To enable Debiased IVW and MRBEE to work with a large number of SNPs, we modified their source code to avoid memory usage problems, by implementing matrix multiplications without generating large intermediate matrices.

Table 5 gives causal estimates from standard IVW MR, without considering selection of disease cases, and MVIVW adjusting for selection but not weak instrument bias. The table also gives estimates from other methods adjusting for selection and weak instrument bias. While the point estimates from simple IVW and MVIVW are negative, consistent with smoking initiation leading to poor prognosis, the estimates with weak instrument adjustments are positive. Estimates from CWLS, MV‐LIML, Debiased IVW and MRBEE are reassuringly similar, with MR‐GRAPPLE producing a smaller estimate. The over‐dispersion parameter in Debiased‐IVW is small, suggesting that the instruments are valid, and explains the concordance between MV‐LIML and the other methods. We note that the standard error from MV‐LIML is apparently underestimated, producing a significantly positive estimate, whereas those from the other methods indicate no robust evidence for a causal effect of smoking initiation on the odds of Crohn's disease good/poor prognosis.

**Table 5:** Estimates of the causal effect of smoking initiation on Crohn's disease prognosis (binary phenotype, good/poor). Standard errors are shown in parentheses.

Exposure	IVW	MVIVW	CWBLS	MV‐LIML	MR‐GRAPPLE	Debiased IVW	MRBEE
Smoking initiation	−0.0175 (0.0416)	−0.0147 (0.0416)	0.1432 (0.2789)	0.1400 (0.0430)	0.1136 (0.2612)	0.1432 (0.2573)	0.1432 (0.2612)

We then estimated the causal association between smoking cessation and Crohn's disease prognosis. Firstly, we needed to correct for collider bias in the smoking cessation effects, as the cessation phenotype is only defined on ever smokers (Liu et al. 2019). As previously discussed (Cai et al. 2022), we adjusted smoking cessation using smoking initiation in instrument effect regression to correct for collider bias and apply CWLS/MR‐RAPS to remove weak instrument bias, based on the same pruned set of SNPs as above. The adjusted summary statistics for smoking cessation were then used to estimate the causal association with Crohn's disease prognosis. Estimates of causal effects are given in Table 6. It is clear that there is little difference whether collider bias adjustment for smoking cessation is performed by CWLS or MR‐RAPS. All estimates from bias‐adjusted methods are positive, suggesting that given Crohn's disease incidence, the log odds of good prognosis increases by approximately 0.9, when the log odds of quitting smoking increases by 1. This suggests that quitters are more likely to achieve good prognosis. However, the standard errors are relatively large and indicate no robust evidence for a causal effect of smoking cessation. Again, the standard error from MV‐LIML appears to be under‐estimated.

**Table 6:** Estimates of the causal effect of smoking cessation on Crohn's disease prognosis (binary phenotype, good/poor). Standard errors are shown in parentheses. Adjustment, method for adjusting smoking cessation effects for collider bias by selection of ever smokers.

Exposure	Adjustment	IVW	MVIVW	CWBLS	MV‐LIML	MR‐GRAPPLE	Debiased IVW	MRBEE
Smoking cessation	CWLS	0.0066 (0.0301)	0.0086 (0.0301)	0.9046 (0.8229)	1.0100 (0.0314)	0.9178 (0.8061)	0.9046 (0.8203)	0.9046 (0.8188)
	MR‐RAPS	0.0072 (0.0297)	0.0090 (0.0297)	0.8633 (0.9562)	0.9660 (0.0310)	0.8808 (0.8009)	0.8633 (0.8143)	0.8633 (0.8128)

## 4 Discussion

In this paper, we have introduced a practical extension of instrument effect regression using two‐sample multivariable MR to estimate the causal effect of a risk factor on disease progression. We showed that the linear structure using summary statistics does not change, with the coefficient of the exposure trait as the causal effect of our interest.

Furthermore, we have proposed CWBLS as a novel correction for weak instrument bias in bivariate MR. We compared the point estimates and coverage with those from multivariable IVW, which is vulnerable to weak instrument bias. We found that generalised instrument effect regression with CWBLS adjustment is a robust method which significantly improves the coverage rate in estimating the causal effect. In case of invalid instruments, CWBLS demonstrates better performance compared to multivariate IVW and MV‐LIML and very similar robust results to MR‐GRAPPLE, Debiased IVW and MRBEE.

A possible advantage of the CWBLS approach is computational efficiency and robustness. In the Supporting Information, we illustrate the run time of an example data set using different methods, while in the Crohn's disease study, we found MR‐GRAPPLE was slow when using more than 100,000 SNPs, a typical number after pruning GWAS data. We also found that MRBEE and Debiased IVW could suffer from memory usage problems with large numbers of SNPs, while MR‐GRAPPLE could suffer bias under a large pleiotropic variance. These issues did not affect CWBLS. In the simulation results, it is clear that the standard errors of CWBLS are almost identical to MR‐GRAPPLE, Debiased IVW and MRBEE, without bias in the estimates. Thus, we can confirm that CWBLS is as robust as other bias‐correction methods in the disease progression scenario to adjust for collider bias and weak instrument bias.

Though our simulations show good performance of CWBLS, we also note some limitations. Firstly, we were unable to obtain an analytical standard error for the CWBLS adjustment. In this paper, we use non‐parametric bootstrapping to estimate the standard error of CWBLS for simplicity. Second, using correlated genetic instrument variables can potentially bias the estimates from CWBLS, due to the violation of the InCLUDE assumption (Cai et al. 2022). In this paper, we have assumed that the IV SNPs are independent, while other methods are not restricted to this assumption. A PCA could be combined with MV‐LIML to select independent instruments, while MR‐GRAPPLE, Debiased IVW and MRBEE can be generalised by using an estimated correlation matrix of instruments. Hence, we suggest selecting independent SNPs by using linkage disequilibrium pruning with a high quality threshold or applying PCA on the variance‐covariance matrix of summary statistics. Note that this assumption is feasible for genome‐wide instruments but less so in *cis‐*MR which focusses on molecular‐level exposures (Batool et al. 2022). However, weak instruments may be less of a problem in that situation, in which case the generalised instrument effect regression using bivariate MR could be used. Finally, CWLS generalisation becomes more complex when there are more than two risk factors. It is worth noting that in the univariate case, CWLS is identical to Debiased IVW (Ye, Shao, and Kang 2019). CWBLS does differ from the bivariate Debiased IVW, but in our simulations their results were almost identical on the average.

While we have focussed on disease progression, any factor associated with the selection of study participants can potentially introduce collider bias (Paternoster et al. 2017). As in previous work for observational associations (Cai et al. 2022), our approach for MR can be applied more generally when subjects are selected or covariates are conditioned upon. The latter has been previously demonstrated in simulations, but without theoretical justification or consideration of weak instruments (Gilbody et al. 2022).

By working with the marginal genetic effects on exposure, as usually obtained from GWAS, we allow the genetic effect on exposure to depend on disease status. However we have only considered the specific form $\beta_{\mathrm{GX}}^{M}=\beta_{\mathrm{GX}}+\beta_{\mathrm{DX}}\beta_{\mathrm{GD}}$, and in general different SNPs may modify their effects on exposure in different ways in the presence of disease. This could be encoded with SNP‐specific interaction terms, but they would not be identifiable in the two‐sample scenarios that usually pertain. We can view our $\beta_{\mathrm{DX}}$ parameter as modelling an average effect of gene‐disease interaction on exposure.

We showed the feasibility of applying our methods to real data by studying the effects of smoking behaviour on Crohn's disease prognosis. Although we did not observe robust evidence for causal effects, the point estimates for smoking cessation were strong, which may encourage current smokers to quit on the diagnosis of Crohn's disease. We have found similar results regarding various types of Crohn's disease progression in other studies, supporting our interpretation (Song et al. 2018; Cosnes et al. 2001; Lunney et al. 2015; Hua et al. 2022).

In conclusion, we use bivariable MR to represent the relationship between risk factors and a disease progression outcome. To deal with weak instrument bias a generalised instrument effect regression model combined with CWBLS adjustment is an effective method to estimate the causal effect, subject to the independence assumptions discussed. These assumptions may be varied by using other MR methods in the bivariate approach.

## Supporting information

Supporting information.

## Data Availability

An open‐source R package implementing the 2‐step methods proposed in this paper is available from https://github.com/SiyangCai/ColliderBias. The data that support the findings of this study are available in the European Genome‐phenome Archive at https://doi.org/10.1038/ng.3755. These data were derived from the following resources available in the public domain: Smoking initiation and cessation, https://conservancy.umn.edu/handle/11299/201564 and Crohn's disease prognosis, https://doi.org/10.1038/ng.3755.
