# Supplementary material for: Estimating Causal Effects on a Disease Progression Trait Using Bivariate Mendelian Randomisation
Source: Genet Epidemiol. 2024 Oct 24;49(1):e22600. doi: 10.1002/gepi.22600 (PMC11656144; doi:10.1002/gepi.22600)
Supplement: Supplementary file 1 — Supporting information. [file GEPI-49-0-s001.docx]

Supplementary material

Contents

[Supplementary material 1](#_Toc172114448)

[Proof of Generalised instrument effect regression 2](#_Toc172114449)

[No correlation between $\boldsymbol{X}$ and $\boldsymbol{D}$ 2](#_Toc172114450)

[Causal effect from $\boldsymbol{X}$ to $\boldsymbol{D}$ 3](#_Toc172114451)

[Causal effect from $\boldsymbol{D}$ to $\boldsymbol{X}$ 6](#_Toc172114452)

[Corrected Weighted Bivariate Least Squares 7](#_Toc172114453)

[R package: *ColliderBias* 7](#_Toc172114454)

[Tables 8](#_Toc172114455)

[References 13](#_Toc172114456)

# Proof of Generalised instrument effect regression

In this section we will provide proof of generalised instrument effect regression in the main paper. We will start with no correlation between exposure and disease trait.

## No correlation between $\boldsymbol{X}$ and $\boldsymbol{D}$

We have exposure:

$$X= \beta_{GX}G+ \beta_{UX}U_{X}+ E_{X},$$

disease trait:

$D= \beta_{GD}G+ \beta_{UD}U_{D}+E_{D}$,

Also the outcome

$Y=\beta_{GY}G+\beta_{UY}U_{Y}+\beta_{DY}D+\beta_{XY}X+E_{Y}$*,*

where $U_{D}=u_{G}+u_{d}$, $U_{X}=u_{G}+u_{x}$, $U_{Y}=u_{G}+u_{x}+u_{d}$.

If $\beta_{\mathrm{GY}}^{'}$ are obtained by $G$ on $Y$ conditional on $D$ from a linear regression [1]:

$\beta_{GY}^{C'}=\frac{1}{var\left( G \right)var\left( D \right)-{cov\left( G,D \right)}^{2}}\left( var\left( D \right)cov\left( G,Y \right)-cov\left( G,D \right)cov\left( D,Y \right) \right)$*.*

From the above equations for $D$ and $Y$:

$$var\left( D \right)=\beta_{GD}^{2}var\left( G \right)+\beta_{UD}^{2}{var(U}_{D})+var\left( E_{D} \right)$$

$$cov\left( G,D \right)=\beta_{GD}var\left( G \right)$$

$$cov\left( G,Y \right)=\beta_{GY}var\left( G \right)+\beta_{DY}\beta_{GD}var\left( G \right)+\beta_{XY}\beta_{GX}var\left( G \right)$$

Because $U_{Y}$ has a genetic confounder, and two non-genetic confounders from $X$ and $D$, we have:

$$cov(D, Y) =\beta_{GD}\beta_{GY}var(G)+ \beta_{UY}\beta_{UD}{var(U}_{D}) + \beta_{DY}var(D) + \beta_{XY}\beta_{GD}\beta_{GX}var(G) + \beta_{XY}\beta_{UX}\beta_{UD} var(u_{G})$$

$= (\beta_{GD}\beta_{GY} + {\beta_{DY}\beta}_{GD}^{2}+ \beta_{XY}\beta_{GD}\beta_{GX}) var\left( G \right)+ (\beta_{UY}\beta_{UD} + \beta_{DY}\beta_{UD}^{2}) {var(U}_{D}) + \beta_{DY}var\left( E_{D} \right) + \beta_{XY}\beta_{UX}\beta_{UD} var(u_{G})$*.*

Then we have:

$$var(D)cov(G,Y)= var(G)(\beta_{GY}+\beta_{DY}\beta_{GD}+\beta_{XY}\beta_{GX})(\beta_{GD}^{2}var\left( G \right)+\beta_{UD}^{2}{var(U}_{D})+var\left( E_{D} \right))$$

$$cov\left( G,D \right)cov\left( D,Y \right) = \beta_{GD}var\left( G \right) [(\beta_{GD}\beta_{GY} + {\beta_{DY}\beta}_{GD}^{2}+ \beta_{XY}\beta_{GD}\beta_{GX}) var\left( G \right)+ (\beta_{UY}\beta_{UD} + \beta_{DY}\beta_{UD}^{2}) {var(U}_{D}) + \beta_{DY}var\left( E_{D} \right) + \beta_{XY}\beta_{UX}\beta_{UD} var(u_{G})]$$

$$var\left( G \right)var\left( D \right)-{cov\left( G,D \right)}^{2}= {var\left( G \right)[\beta}_{UD}^{2}{var(U}_{D})+var\left( E_{D} \right)]$$

Rearrange and we have:

$$\beta_{GY}^{C'}=\beta_{GY}+\beta_{XY}\beta_{GX}+\frac{-\beta_{UY}\beta_{UD}var\left( U_{D} \right)-\beta_{XY}\beta_{UX}\beta_{UD}var\left( u_{G} \right)}{\beta_{UD}^{2}{var(U}_{D})+var\left( E_{D} \right)}\beta_{GD}$$

## Causal effect from $\boldsymbol{X}$ to $\boldsymbol{D}$

We have exposure:

$$X= \beta_{GX}G+ \beta_{UX}U_{X}+ E_{X},$$

disease trait:

$D=\beta_{GD}G+\beta_{UD}U_{D}+E_{D}+\beta_{XD}X$*,*

Also the outcome

$Y=\beta_{GY}G+\beta_{UY}U_{Y}+\beta_{DY}D+\beta_{XY}X+E_{Y}$*,*

where $U_{D}=u_{G}+u_{d}$, $U_{X}=u_{G}+u_{x}$, $U_{Y}=u_{G}+u_{x}+u_{d}$.

As above, we have

$\beta_{GY}^{C'}=\frac{1}{var\left( G \right)var\left( D \right)-{cov\left( G,D \right)}^{2}}\left( var\left( D \right)cov\left( G,Y \right)-cov\left( G,D \right)cov\left( D,Y \right) \right)$*.*

We now have

$$var\left( D \right)=\beta_{GD}^{2}var\left( G \right)+\beta_{UD}^{2}var\left( U_{D} \right)+var\left( E_{D} \right)+\beta_{XD}^{2}\left( \beta_{GX}^{2}var\left( G \right)+\beta_{UX}^{2}var\left( U_{X} \right)+var\left( E_{X} \right) \right)+2\beta_{XD}cov\left( X,\beta_{GD}G+\beta_{UD}U_{D} \right)$$

Where:

$$2\beta_{XD}cov\left( X,\beta_{GD}G+\beta_{UD}U_{D} \right)=2\left( \beta_{XD}\beta_{GD}\beta_{GX}var\left( G \right)+\beta_{XD}\beta_{UD}\beta_{UX}var\left( u_{G} \right) \right)$$

Also:

$$cov\left( G,D \right)=\beta_{GD}var\left( G \right)+\beta_{XD}\beta_{GX}var\left( G \right)$$

And:

$$cov\left( G,Y \right)=\beta_{GY}var\left( G \right)+\beta_{DY}\beta_{GD}var\left( G \right)+\beta_{XY}\beta_{GX}var\left( G \right)+\beta_{DY}\beta_{XD}\beta_{GX}var\left( G \right)$$

Finally, we have:

$$cov\left( D,Y \right)=\beta_{GY}cov\left( G,D \right)+\beta_{DY}cov\left( D,D \right)+\beta_{XY}cov\left( D,X \right)+\beta_{UY}cov\left( D,U_{Y} \right)$$

Where:

$$\beta_{GY}cov\left( G,D \right)=\beta_{GY}(\beta_{GD}+\beta_{XD}\beta_{GX})var\left( G \right)$$

$$\beta_{DY}cov\left( D,D \right)=\beta_{DY}var\left( D \right)$$

$$\beta_{XY}cov\left( D,X \right)=\left( \beta_{GD}+\beta_{XD}\beta_{GX} \right)\beta_{XY}\beta_{GX}var\left( G \right)+\beta_{XY}\beta_{XD}\beta_{UX}^{2}var\left( U_{X} \right)+\beta_{XY}\beta_{UX}\beta_{UD}var\left( u_{G} \right)$$

$$\beta_{UY}cov\left( D,U_{Y} \right)=\beta_{UY}\beta_{UD}var\left( U_{D} \right)+\beta_{UY}\beta_{XD}\beta_{UX}var\left( U_{X} \right)$$

Here we have the denominator:

$$var\left( G \right)var\left( D \right)-cov\left( D,G \right)^{2}=\left( \beta_{UD}^{2}var\left( U_{D} \right)+\beta_{XD}^{2}\beta_{UX}^{2}var\left( U_{X} \right)+var\left( E_{D} \right)+\beta_{XD}^{2}var\left( E_{X} \right)+ 2\beta_{XD}\beta_{UD}\beta_{UX}var\left( u_{G} \right) \right)var\left( G \right)$$

And the numerator:

$$var\left( D \right)cov\left( G,Y \right)=\left[ \beta_{GD}^{2}var\left( G \right)+\beta_{UD}^{2}var\left( U_{D} \right)+var\left( E_{D} \right)+\beta_{XD}^{2}\left( \beta_{GX}^{2}var\left( G \right)+\beta_{UX}^{2}var\left( U_{X} \right)+var\left( E_{X} \right) \right)+ 2\left( \beta_{XD}\beta_{GD}\beta_{GX}var\left( G \right)+\beta_{XD}\beta_{UD}\beta_{UX}var\left( u_{G} \right) \right) \right]\left( \beta_{GY}+\beta_{DY}\beta_{GD}+\beta_{XY}\beta_{GX}+\beta_{DY}\beta_{XD}\beta_{GX} \right)var\left( G \right)$$

$$cov\left( G,D \right)cov\left( D,Y \right)=\left( \beta_{GY}cov\left( G,D \right)+\beta_{DY}var(D)+\beta_{XY}cov\left( D,X \right)+\beta_{UY}cov\left( D,U_{Y} \right) \right)\left( \beta_{GD}+\beta_{XD}\beta_{GX} \right)var\left( G \right)$$

Comparing both terms, we can eliminate $\left( \beta_{GD}+\beta_{XD}\beta_{GX} \right)var\left( G \right)\beta_{DY}var\left( D \right)$. This gives:

$$var\left( D \right)cov\left( G,Y \right)=\left[ \beta_{GD}^{2}var\left( G \right)+\beta_{UD}^{2}var\left( U_{D} \right)+var\left( E_{D} \right)+\beta_{XD}^{2}\left( \beta_{GX}^{2}var\left( G \right)+\beta_{UX}^{2}var\left( U_{X} \right)+var\left( E_{X} \right) \right)+ 2\left( \beta_{XD}\beta_{GD}\beta_{GX}var\left( G \right)+\beta_{XD}\beta_{UD}\beta_{UX}var\left( u_{G} \right) \right) \right]\left( \beta_{GY}+\beta_{XY}\beta_{GX} \right)var\left( G \right)+\left( \beta_{GD}+\beta_{XD}\beta_{GX} \right)var\left( G \right)\beta_{DY}var\left( D \right)$$

$$cov\left( G,D \right)cov\left( D,Y \right)=\left( \beta_{GY}cov\left( G,D \right)+\beta_{XY}cov\left( D,X \right)+\beta_{UY}cov\left( D,U_{Y} \right) \right)\left( \beta_{GD}+\beta_{XD}\beta_{GX} \right)var\left( G \right)+\left( \beta_{GD}+\beta_{XD}\beta_{GX} \right)var\left( G \right)\beta_{DY}var\left( D \right)$$

$$=\left( \beta_{GY}\beta_{GD}^{2}+\beta_{GY}\beta_{XD}^{2}\beta_{GX}^{2}+2{\beta_{GY}\beta}_{GD}\beta_{XD}\beta_{GX} \right)var^{2}\left( G \right)+\left[ \left( \beta_{GD}+\beta_{XD}\beta_{GX} \right)\beta_{XY}\beta_{GX}var\left( G \right)+\beta_{XY}\beta_{XD}\beta_{UX}^{2}var\left( U_{X} \right)+\beta_{XY}\beta_{UX}\beta_{UD}var\left( u_{G} \right)+\beta_{UY}\beta_{UD}var\left( U_{D} \right)+\beta_{UY}\beta_{XD}\beta_{UX}var\left( U_{X} \right) \right] \left( \beta_{GD}+\beta_{XD}\beta_{GX} \right)var\left( G \right)+\left( \beta_{GD}+\beta_{XD}\beta_{GX} \right)var\left( G \right)\beta_{DY}var\left( D \right)$$

Again, comparing both terms, we are able to eliminate $\left( \beta_{GY}\beta_{GD}^{2}+\beta_{GY}\beta_{XD}^{2}\beta_{GX}^{2} \right) var^{2}\left( G \right) +2\beta_{GY}\beta_{GD}\beta_{XD}\beta_{GX}var^{2}\left( G \right)$, which leaves us with:

$$var\left( D \right)cov\left( G,Y \right)=\left[ \beta_{UD}^{2}var\left( U_{D} \right)+var\left( E_{D} \right)+\beta_{XD}^{2}\left( \beta_{UX}^{2}var\left( U_{X} \right)+var\left( E_{X} \right) \right)+ 2\left( \beta_{XD}\beta_{UD}\beta_{UX}var\left( u_{G} \right) \right) \right]\left( \beta_{GY}+\beta_{XY}\beta_{GX} \right)var\left( G \right)+\left( \beta_{XY}\beta_{GX}\beta_{GD}^{2}+\beta_{XY}\beta_{XD}^{2}\beta_{GX}^{3} \right)var^{2}\left( G \right)+2{\beta_{XY}\beta}_{XD}\beta_{GD}\beta_{GX}^{2}var^{2}\left( G \right)$$

$$cov\left( G,D \right)cov\left( D,Y \right)=\left[ \left( \beta_{GD}+\beta_{XD}\beta_{GX} \right)\beta_{XY}\beta_{GX}var\left( G \right)+\beta_{XY}\beta_{XD}\beta_{UX}^{2}var\left( U_{X} \right)+\beta_{XY}\beta_{UX}\beta_{UD}var\left( u_{G} \right)+\beta_{UY}\beta_{UD}var\left( U_{D} \right)+\beta_{UY}\beta_{XD}\beta_{UX}var\left( U_{X} \right) \right] \left( \beta_{GD}+\beta_{XD}\beta_{GX} \right)var\left( G \right)$$

Comparing with denominator$var\left( G \right)var\left( D \right)-cov\left( D,G \right)^{2}$, we can get $\beta_{GY}+\beta_{XY}\beta_{GX}$ outside of the fraction, which leaves us with:

$$var\left( D \right)cov\left( G,Y \right) = \left( \beta_{XY}\beta_{GX}\beta_{GD}^{2}+\beta_{XY}\beta_{XD}^{2}\beta_{GX}^{3} \right)var^{2}\left( G \right)+2{\beta_{XY}\beta}_{XD}\beta_{GD}\beta_{GX}^{2}var^{2}\left( G \right)$$

$$cov\left( G,D \right)cov\left( D,Y \right)=\left[ \left( \beta_{GD}+\beta_{XD}\beta_{GX} \right)\beta_{XY}\beta_{GX}var\left( G \right)+\beta_{XY}\beta_{XD}\beta_{UX}^{2}var\left( U_{X} \right)+\beta_{UY}\beta_{UD}var\left( U_{D} \right)+\beta_{UY}\beta_{XD}\beta_{UX}var\left( U_{X} \right) \right] \left( \beta_{GD}+\beta_{XD}\beta_{GX} \right)var\left( G \right)$$

$$=\left[ \beta_{XY}\beta_{XD}\beta_{UX}^{2}var\left( U_{X} \right)+\beta_{UY}\beta_{UD}var\left( U_{D} \right)+\beta_{UY}\beta_{XD}\beta_{UX}var\left( U_{X} \right)+\beta_{XY}\beta_{UX}\beta_{UD}var\left( u_{G} \right) \right]\left( \beta_{GD}+\beta_{XD}\beta_{GX} \right)var\left( G \right)+\left( \beta_{XY}\beta_{GX}\beta_{GD}^{2}+2{\beta_{XY}\beta}_{XD}\beta_{GD}\beta_{GX}^{2}+\beta_{XY}\beta_{XD}^{2}\beta_{GX}^{3} \right)var^{2}\left( G \right)$$

So that we can get rid of $\left( \beta_{XY}\beta_{GX}\beta_{GD}^{2}+2{\beta_{XY}\beta}_{XD}\beta_{GD}\beta_{GX}^{2}+\beta_{XY}\beta_{XD}^{2}\beta_{GX}^{3} \right)var^{2}\left( G \right)$

Then the new instrumental effect regression becomes:

$$\beta_{GY}^{C^{'}}=\beta_{GY}+\beta_{XY}\beta_{GX}++\frac{- \left[ \beta_{XY}\beta_{XD}\beta_{UX}^{2}var\left( U_{X} \right)+\beta_{UY}\beta_{UD}var\left( U_{D} \right)+\beta_{UY}\beta_{XD}\beta_{UX}var\left( U_{X} \right)+\beta_{XY}\beta_{UX}\beta_{UD}var\left( u_{G} \right) \right]numerator}{\left( \beta_{UD}^{2}var\left( U_{D} \right)+\beta_{XD}^{2}\beta_{UX}^{2}var\left( U_{X} \right)+ 2\beta_{XD}\beta_{UD}\beta_{UX}var\left( u_{G} \right)+var\left( E_{D} \right)+\beta_{XD}^{2}var\left( E_{X} \right) \right)}\left( \beta_{GD}+\beta_{XD}\beta_{GX} \right)$$

$$numerator= - \left[ \beta_{XY}\beta_{XD}\beta_{UX}^{2}var\left( U_{X} \right)+\beta_{UY}\beta_{UD}var\left( U_{D} \right)+\beta_{UY}\beta_{XD}\beta_{UX}var\left( U_{X} \right)+\beta_{XY}\beta_{UX}\beta_{UD}var\left( u_{G} \right) \right]\left( \beta_{GD}+\beta_{XD}\beta_{GX} \right)$$

This is the generalised instrumental effect regression with marginal genetic effects $\beta_{GD}^{M}=\beta_{GD}+\beta_{GX}\beta_{XD}$. When $\beta_{XD} = 0$, this reduces to previous instrumental effect regression.

## Causal effect from $\boldsymbol{D}$ to $\boldsymbol{X}$

We have exposure:

$$X= \beta_{GX}G+ \beta_{UX}U_{X}+ E_{X} +\beta_{DX}{+ \beta}_{DX}D,$$

disease trait:

$D= \beta_{GD}G+ \beta_{UD}U_{D}+E_{D}$,

Also the outcome

$Y=\beta_{GY}G+\beta_{UY}U_{Y}+\beta_{DY}D+\beta_{XY}X+E_{Y}$*,*

where $U_{D}=u_{G}+u_{d}$, $U_{X}=u_{G}+u_{x}$, $U_{Y}=u_{G}+u_{x}+u_{d}$.

Again, we have

$\beta_{GY}^{C'}=\frac{1}{var\left( G \right)var\left( D \right)-{cov\left( G,D \right)}^{2}}\left( var\left( D \right)cov\left( G,Y \right)-cov\left( G,D \right)cov\left( D,Y \right) \right)$*.*

To derive the terms:

$$var\left( D \right)=\beta_{GD}^{2}var\left( G \right)+\beta_{UD}^{2}{var(U}_{D})+var\left( E_{D} \right)$$

$$cov\left( G,D \right)=\beta_{GD}var\left( G \right)$$

However, the other two terms are slightly different:

$$cov\left( G,Y \right)=\beta_{GY}var\left( G \right)+\beta_{DY}\beta_{GD}var\left( G \right)+\beta_{XY}\beta_{GX}var\left( G \right) + \beta_{XY}\beta_{DX}\beta_{GD}var\left( G \right)$$

And:

$$cov(D, Y) =\beta_{GD}\beta_{GY}var(G)+ \beta_{UY}\beta_{UD}{var(U}_{D}) + \beta_{DY}var(D)+ \beta_{XY}cov(X, D)$$

Where:

$$cov\left( X,D \right)=\beta_{GX}\beta_{GD}var\left( G \right)+ \beta_{UX}\beta_{UD} var(u_{G})+\beta_{DX}var\left( D \right)$$

To construct the formula, here we have in the denominator:

$$var\left( G \right)var\left( D \right)-cov\left( D,G \right)^{2} = {var\left( G \right)[\beta}_{UD}^{2}{var(U}_{D})+var\left( E_{D} \right)]$$

To compare $var\left( D \right)cov\left( G,Y \right)-cov\left( G,D \right)cov\left( D,Y \right)$ with the previous formula, we see the difference is:

$$\beta_{XY}\beta_{XD}\beta_{GD}var\left( G \right)var\left( D \right)-{\beta_{XY}\beta}_{GD}\beta_{XD}var\left( G \right)var\left( D \right)=0$$

So the formula is not changed from the “causal independence” assumption:

$$\beta_{GY}^{C'}=\beta_{GY}+\beta_{XY}\beta_{GX}+\frac{-\beta_{UY}\beta_{UD}var\left( U_{D} \right)-\beta_{XY}\beta_{UX}\beta_{UD}var\left( u_{G} \right)}{\beta_{UD}^{2}{var(U}_{D})+var\left( E_{D} \right)}\beta_{GD}$$

Replacing $\beta_{GX}$ by the marginal effect $\beta_{GX}^{M}=\beta_{GX}+\beta_{GD}\beta_{DX}$, we have the equation (3) in the main paper.

# Corrected Weighted Bivariate Least Squares

Consider an IVW model:

$Y=\beta X+ \epsilon$*,*

with the design matrix $X=\left( \begin{matrix} X^{T} & D^{T} \end{matrix} \right)=\left( \begin{matrix} x_{1} & d_{1} \\ \vdots& \vdots\\ x_{n} & d_{n} \end{matrix} \right)$*,* $Y=\left( \begin{matrix} y_{1} \\ \vdots\\ y_{n} \end{matrix} \right)$ and weights as a diagonal matrix $W = \left( \begin{matrix} w_{1} & \cdots& 0 \\ \vdots& \ddots& \vdots\\ 0 & \cdots& w_{n} \end{matrix} \right)$, where $w_{i}=\sigma_{Y_{i}}^{-2}\frac{1}{\sigma_{Y_{i}}^{2}}$.

Then the slopes from weighted multivariate least squares are:

$\beta=\left( \begin{matrix} \beta_{X} \\ \beta_{D} \end{matrix} \right)$ *=* $\left( X^{T}WX \right)^{-1}X^{T}WY$*,*

where

$$X^{T}WX=\left( \begin{matrix} x_{1} & \ldots& x_{n} \\ d_{1} & \ldots& d_{n} \end{matrix} \right)\left( \begin{matrix} w_{1} & \cdots& 0 \\ \vdots& \ddots& \vdots\\ 0 & \cdots& w_{n} \end{matrix} \right)\left( \begin{matrix} x_{1} & d_{1} \\ \vdots& \vdots\\ x_{n} & d_{n} \end{matrix} \right)=\left( \begin{matrix} \sum w_{i}x_{i}^{2} & \sum w_{i}x_{i}d_{i} \\ \sum w_{i}x_{i}d_{i} & \sum w_{i}d_{i}^{2} \end{matrix} \right)$$

$$\left( X^{T}WX \right)^{-1}=\frac{1}{\sum w_{i}x_{i}^{2}\sum w_{i}d_{i}^{2}-\left( \sum w_{i}x_{i}d_{i} \right)^{2}}\left( \begin{matrix} \sum w_{i}d_{i}^{2} & -\sum w_{i}x_{i}d_{i} \\ -\sum w_{i}x_{i}d_{i} & \sum w_{i}x_{i}^{2} \end{matrix} \right)$$

$$X^{T}WY=\left( \begin{matrix} \sum w_{i}x_{i}y_{i} \\ \sum w_{i}d_{i}y_{i} \end{matrix} \right)$$

This yields:

$$\beta=\left( \begin{matrix} \beta_{XY} \\ \beta_{DY} \end{matrix} \right) = \frac{1}{\sum w_{i}x_{i}^{2}\sum w_{i}d_{i}^{2}-\left( \sum w_{i}x_{i}d_{i} \right)^{2}}\left( \begin{matrix} \sum w_{i}d_{i}^{2} & -\sum w_{i}x_{i}d_{i} \\ -\sum w_{i}x_{i}d_{i} & \sum w_{i}x_{i}^{2} \end{matrix} \right)\left( \begin{matrix} \sum w_{i}x_{i}y_{i} \\ \sum w_{i}d_{i}y_{i} \end{matrix} \right)$$

$$\left( \begin{matrix} \beta_{XY} \\ \beta_{DY} \end{matrix} \right) = \frac{1}{\sum w_{i}x_{i}^{2}\sum w_{i}d_{i}^{2}-\left( \sum w_{i}x_{i}d_{i} \right)^{2}}\left( \begin{matrix} \sum w_{i}d_{i}^{2}\sum w_{i}x_{i}y_{i}- \sum w_{i}x_{i}d_{i}\sum w_{i}d_{i}y_{i} \\ \sum w_{i}x_{i}^{2}\sum w_{i}d_{i}y_{i} - \sum w_{i}x_{i}d_{i}\sum w_{i}x_{i}y_{i} \end{matrix} \right)$$

So we have:

$$\hat{b}=\beta_{XY}{\hat{b}=\beta}_{XY}=\frac{\sum w_{i}d_{i}^{2}\sum w_{i}x_{i}y_{i}- \sum w_{i}x_{i}d_{i}\sum w_{i}d_{i}y_{i}}{\sum w_{i}x_{i}^{2}\sum w_{i}d_{i}^{2}-\left( \sum w_{i}x_{i}d_{i} \right)^{2}},$$

where $x_{i}$ are true genetic effects of $G$ on $X,$ $d_{i}$ are true genetic effects of $G$ on $D$ and $y_{i}$ are true genetic effects of $G$ on $Y$ conditioning on $D$.

Let estimated effects be  $\hat{x}_{i}=x_{i}+\varepsilon_{X_{i}}$ where $\varepsilon_{X_{i}}$ has mean 0 and variance $\sigma_{X_{i}}^{2}$ independently of all $x_{i}$. Similarly for  $\hat{d}_{i}$ and  $\hat{y}_{i}$. Assume that $\varepsilon_{X_{i}}$ and $\varepsilon_{D_{i}}$ are independent, as in a two-sample MR design. Then the biased estimate of $b$ is

$$\hat{b}_{bias}=\frac{\sum w_{i}\hat{d}_{i}^{2}\sum w_{i}\hat{x}_{i}\hat{y}_{i}- \sum w_{i}\hat{x}_{i}\hat{d}_{i}\sum w_{i}\hat{d}_{i}\hat{y}_{i}}{\sum w_{i}\hat{x}_{i}^{2}\sum w_{i}\hat{d}_{i}^{2}-\left( \sum w_{i}\hat{x}_{i}\hat{d}_{i} \right)^{2}},$$

In the numerator:

$$\sum w_{i}\hat{d}_{i}^{2}\sum w_{i}\hat{x}_{i}\hat{y}_{i}=\sum w_{i}\left( d_{i}+\varepsilon_{D_{i}} \right)^{2}\sum w_{i}{(x}_{i}+\varepsilon_{X_{i}})y_{i}\approx\sum w_{i}x_{i}y_{i}\left( \sum w_{i}d_{i}^{2}+\sum w_{i}\varepsilon_{D_{i}}^{2} \right),$$

$$\sum w_{i}\hat{x}_{i}\hat{d}_{i}\sum w_{i}\hat{d}_{i}\hat{y}_{i}=\sum w_{i}{(x}_{i}+\varepsilon_{X_{i}})\left( d_{i}+\varepsilon_{D_{i}} \right)\sum w_{i}y_{i}\left( d_{i}+\varepsilon_{D_{i}} \right)\approx\sum w_{i}x_{i}d_{i}\sum w_{i}y_{i}d_{i} + \sum w_{i}\varepsilon_{X_{i}}\varepsilon_{D_{i}}\sum w_{i}d_{i}y_{i},$$

In the denominator:

$$\sum w_{i}\hat{x}_{i}^{2}\sum w_{i}\hat{d}_{i}^{2}=\sum w_{i}\left( x_{i}+\varepsilon_{X_{i}} \right)^{2}\sum w_{i}\left( d_{i}+\varepsilon_{D_{i}} \right)^{2}\approx\left( \sum w_{i}x_{i}^{2}+\sum w_{i}\varepsilon_{X_{i}}^{2} \right)\left( \sum w_{i}d_{i}^{2}+\sum w_{i}\varepsilon_{D_{i}}^{2} \right)$$

$${\left( \sum w_{i}\hat{x}_{i}\hat{d}_{i} \right)^{2}=\left( \sum w_{i}\left( x_{i}+\varepsilon_{X_{i}} \right)\left( d_{i}+\varepsilon_{D_{i}} \right) \right)}^{2}\approx\left( \sum w_{i}x_{i}d_{i}+\sum w_{i}\varepsilon_{X_{i}}\varepsilon_{D_{i}} \right)^{2}$$

Substituting into $\hat{b}_{bias}$ we have:

$$\hat{b}_{bias}=\frac{\sum w_{i}d_{i}^{2}\sum w_{i}x_{i}y_{i}-\sum w_{i}x_{i}d_{i}\sum w_{i}d_{i}y_{i}+N}{\sum w_{i}x_{i}^{2}\sum w_{i}d_{i}^{2}-\left( \sum w_{i}x_{i}d_{i} \right)^{2}+D},$$

where

$$N=\sum w_{i}\varepsilon_{D_{i}}^{2}\sum w_{i}x_{i}y_{i}-\sum w_{i}\varepsilon_{X_{i}}\varepsilon_{D_{i}}\sum w_{i}d_{i}y_{i},$$

and

$$D=\sum w_{i}\varepsilon_{D_{i}}^{2}\sum w_{i}x_{i}^{2}+\sum w_{i}\varepsilon_{X_{i}}^{2}\sum w_{i}d_{i}^{2}+\sum w_{i}\varepsilon_{X_{i}}^{2}\sum w_{i}\varepsilon_{D_{i}}^{2}-\left( \sum w_{i}\varepsilon_{X_{i}}\varepsilon_{D_{i}} \right)^{2}-2\sum w_{i}x_{i}d_{i}\sum w_{i}\varepsilon_{X_{i}}\varepsilon_{D_{i}},$$

Approximate $\sum w_{i}\varepsilon_{D_{i}}^{2}$ by $\sum w_{i}\sigma_{D_{i}}^{2}$ and $\sum w_{i}\varepsilon_{X_{i}}^{2}$ by $\sum w_{i}\sigma_{X_{i}}^{2}$. From the two-sample independence assumption, $\sum w_{i}\varepsilon_{x_{i}}\varepsilon_{d_{i}}\approx0$. Then the corrected estimate is

$$\hat{b}_{CWBLS}=\frac{\sum w_{i}\hat{d}_{i}^{2}\sum w_{i}\hat{x}_{i}\hat{y}_{i}- \sum w_{i}\hat{x}_{i}\hat{d}_{i}\sum w_{i}\hat{d}_{i}\hat{y}_{i}-N}{\sum w_{i}\hat{x}_{i}^{2}\sum w_{i}\hat{d}_{i}^{2}-\left( \sum w_{i}\hat{x}_{i}\hat{d}_{i} \right)^{2}-D}$$

where

$$N\approx\sum w_{i}\sigma_{D_{i}}^{2}\sum w_{i}x_{i}y_{i},$$

$$D\approx\sum w_{i}\sigma_{D_{i}}^{2}\sum w_{i}x_{i}^{2}+\sum w_{i}\sigma_{X_{i}}^{2}\sum w_{i}d_{i}^{2}+\sum w_{i}\sigma_{X_{i}}^{2}\sum w_{i}\sigma_{D_{i}}^{2}.$$

However, $\hat{b}_{CWBLS}$ is not available as $N$ and $D$ consist of true effects. Note that

$$\sum w_{i}\left( x_{i}+\varepsilon_{X_{i}} \right)^{2}\approx\sum w_{i}x_{i}^{2}+\sum w_{i}\varepsilon_{X_{i}}^{2},$$

which we can approximate by

$$\approx\sum w_{i}\left( x_{i}^{2}+\sigma_{X_{i}}^{2} \right).$$

So

$$\sum w_{i}x_{i}^{2}\approx\sum w_{i}\left( \hat{x}_{i}^{2}-\sigma_{X_{i}}^{2} \right)$$

Similarly

$$\sum w_{i}d_{i}^{2}\approx\sum w_{i}\left( \hat{d}_{i}^{2}-\sigma_{D_{i}}^{2} \right),$$

$$\sum w_{i}x_{i}y_{i}\approx\sum w_{i}\hat{x}_{i}\hat{y}_{i},$$

Therefore

$$N\approx\sum w_{i}\sigma_{d_{i}}^{2}\sum w_{i}\hat{x}_{i}\hat{y}_{i},$$

$$D\approx\sum w_{i}\sigma_{d_{i}}^{2}\sum w_{i}x_{i}^{2}+\sum w_{i}\sigma_{x_{i}}^{2}\sum w_{i}d_{i}^{2}-\sum w_{i}\sigma_{X_{i}}^{2}\sum w_{i}\sigma_{D_{i}}^{2}$$

which only requires the estimates from $\hat{x}_{i}, \hat{d}_{i}$, $\hat{d}_{i}$ and $\hat{y}_{i}$ and corresponding standard errors.

# Supplementary Tables

Here are a list of tables cited in the main paper.

| $\left( h_{GX}^{2},h_{GD}^{2} \right)H_{GX}$ $H_{GD}$ | $\boldsymbol{\beta}_{\boldsymbol{XY}}$ | MVIVW | | | | MV-LIML | | | | CWBLS | | | | MR-GRAPPLE | | | | Debias IVW | | | | MRBEE | | | |
| --- | --- | --- | --- | --- | --- | --- | --- | --- | --- | --- | --- | --- | --- | --- | --- | --- | --- | --- | --- | --- | --- | --- | --- | --- | --- |
|  |  | EST | CVG | SE | SD | EST | CVG | SE | SD | EST | CVG | SE | SD | EST | CVG | SE | SD | EST | CVG | SE | SD | EST | CVG | SE | SD |
| (0.3, 0.3) | 0.4 | 0.1501 | 0 | 0.0084 | 0.0084 | 0.4002 | 56.2 | 0.0090 | 0.0269 | 0.4002 | 94.9 | 0.0253 | 0.0244 | 0.3960 | 96.4 | 0.0249 | 0.0236 | 0.4002 | 95.4 | 0.0252 | 0.0241 | 0.4002 | 95.6 | 0.0252 | 0.0239 |
| (0.3, 0.1) |  | 0.1501 | 0 | 0.0084 | 0.0083 | 0.4001 | 55.8 | 0.0091 | 0.0254 | 0.3998 | 95.3 | 0.0254 | 0.0244 | 0.3955 | 95.9 | 0.0250 | 0.0228 | 0.3998 | 95.4 | 0.0253 | 0.0244 | 0.3934 | 91.2 | 0.0252 | 0.0271 |
| (0.1, 0.3) |  | 0.0669 | 0 | 0.0097 | 0.0097 | 0.4011 | 27.4 | 0.0105 | 0.0586 | 0.4024 | 96.2 | 0.0684 | 0.0651 | 0.3996 | 96.3 | 0.0647 | 0.0579 | 0.4024 | 95.9 | 0.0670 | 0.0651 | 0.4028 | 93.1 | 0.0624 | 0.0679 |
| (0.1, 0.1) |  | 0.0669 | 0 | 0.0097 | 0.0097 | 0.4011 | 27.8 | 0.0105 | 0.0587 | 0.4031 | 96.3 | 0.0691 | 0.0654 | 0.3998 | 96.4 | 0.065 | 0.0580 | 0.4031 | 95.9 | 0.0674 | 0.0654 | 0.4033 | 95.9 | 0.0676 | 0.0655 |
| (0.3, 0.3) | 0 | 0.0001 | 94.7 | 0.0077 | 0.0077 | 0.0006 | 54.0 | 0.0079 | 0.0209 | 0.0008 | 96.1 | 0.0212 | 0.0209 | 0.0007 | 95.9 | 0.0210 | 0.0207 | 0.0008 | 95.7 | 0.0211 | 0.0209 | 0.0008 | 95.9 | 0.0212 | 0.0210 |
| (0.3, 0.1) |  | 0.0001 | 94.8 | 0.0077 | 0.0077 | 0.0006 | 55.0 | 0.0078 | 0.0290 | 0.0008 | 96.0 | 0.0213 | 0.0215 | 0.0008 | 96.0 | 0.0210 | 0.0209 | 0.0008 | 95.9 | 0.0212 | 0.0211 | 0.0003 | 99.7 | 0.0209 | 0.0211 |
| (0.1, 0.3) |  | 0.0001 | 93.9 | 0.0089 | 0.0089 | 0.0014 | 25.9 | 0.0091 | 0.0183 | 0.0022 | 95.9 | 0.0562 | 0.0540 | 0.0021 | 95.4 | 0.0540 | 0.051 | 0.0022 | 95.5 | 0.0555 | 0.055 | 0.0023 | 95.7 | 0.0556 | 0.056 |
| (0.1, 0.1) |  | 0.0001 | 94.1 | 0.0089 | 0.0089 | 0.0013 | 26.5 | 0.0092 | 0.055 | 0.0023 | 96.3 | 0.0568 | 0.0552 | 0.0021 | 95.5 | 0.0542 | 0.0534 | 0.0023 | 95.7 | 0.0558 | 0.0552 | 0.0011 | 100 | 0.0549 | 0.0273 |
| (0.3, 0.3) | -0.4 | -0.1500 | 0 | 0.0071 | 0.0072 | -0.3993 | 56.4 | 0.0078 | 0.0236 | -0.4015 | 95.3 | 0.0222 | 0.0220 | -0.3973 | 96.5 | 0.0220 | 0.0215 | -0.4015 | 95.8 | 0.0222 | 0.0217 | -0.4016 | 96.0 | 0.0222 | 0.0224 |
| (0.3, 0.1) |  | -0.1499 | 0 | 0.0071 | 0.0071 | -0.3993 | 55.8 | 0.0079 | 0.0197 | -0.4007 | 95.6 | 0.0224 | 0.0217 | -0.3963 | 96.3 | 0.0220 | 0.0200 | -0.4007 | 95.3 | 0.0222 | 0.0217 | -0.3969 | 94.1 | 0.0222 | 0.0231 |
| (0.1, 0.3) |  | -0.0666 | 0 | 0.0082 | 0.0083 | -0.3994 | 27.9 | 0.0092 | 0.0499 | -0.4064 | 95.6 | 0.0602 | 0.0587 | -0.3978 | 96.2 | 0.0571 | 0.0510 | -0.4064 | 95.7 | 0.0594 | 0.0587 | -0.4067 | 95.5 | 0.0595 | 0.0587 |
| (0.1, 0.1) |  | -0.0666 | 0 | 0.0082 | 0.0083 | -0.3994 | 28.2 | 0.0092 | 0.0499 | -0.4071 | 95.9 | 0.0608 | 0.0588 | -0.3992 | 96.2 | 0.0573 | 0.0510 | -0.4071 | 95.7 | 0.0597 | 0.0588 | -0.4074 | 95.5 | 0.0599 | 0.0589 |

Supplementary Table 1: Results from the simulation study of multivariable IVW (MVIVW), MV-LIML, CWBLS, MR-GRAPPLE, Debiased IVW and MRBEE to estimate the causal effect on disease progression, without causal relationship between exposure of interest and disease liability. $h_{GX}^{2}$ and $h_{GD}^{2}$ are heritabilities of the exposure and disease trait. $\beta_{XY}$ is the true causal effect between the exposure and disease progression. EST, mean estimate over 1000 simulations; CVG, coverage of 95% confidence interval; SE, mean of estimated standard errors; SD, empirical standard deviation of estimates. $H_{GX}$ $H_{GD}$ $\beta_{XY}$ Disease trait is simulated as a continuous trait.

| $\left( h_{GX}^{2},h_{GD}^{2} \right)H_{GX}$ $H_{GD}$ | $\boldsymbol{\beta}_{\boldsymbol{XY}}$ | MVIVW | | | | MV-LIML | | | | CWBLS | | | | MR-GRAPPLE | | | | Debias IVW | | | | MRBEE | | | |
| --- | --- | --- | --- | --- | --- | --- | --- | --- | --- | --- | --- | --- | --- | --- | --- | --- | --- | --- | --- | --- | --- | --- | --- | --- | --- |
|  |  | EST | CVG | SE | SD | EST | CVG | SE | SD | EST | CVG | SE | SD | EST | CVG | SE | SD | EST | CVG | SE | SD | EST | CVG | SE | SD |
| (0.3, 0.3) | 0.4 | 0.1497 | 0 | 0.0084 | 0.0087 | 0.4002 | 56.2 | 0.0091 | 0.0239 | 0.4007 | 95.1 | 0.0251 | 0.0254 | 0.3966 | 95.7 | 0.0249 | 0.0241 | 0.4007 | 95.5 | 0.0251 | 0.0254 | 0.4008 | 95.4 | 0.0252 | 0.0255 |
| (0.3, 0.1) |  | 0.1497 | 0 | 0.0084 | 0.0085 | 0.4001 | 55.8 | 0.0091 | 0.0233 | 0.4005 | 95.6 | 0.0254 | 0.0249 | 0.3961 | 96.0 | 0.0250 | 0.0236 | 0.4005 | 95.3 | 0.0253 | 0.0249 | 0.4006 | 95.3 | 0.0253 | 0.0249 |
| (0.1, 0.3) |  | 0.0665 | 0 | 0.0097 | 0.0097 | 0.4011 | 27.4 | 0.0105 | 0.0583 | 0.4033 | 97.0 | 0.0678 | 0.0645 | 0.3982 | 97.0 | 0.0644 | 0.0582 | 0.4033 | 96.6 | 0.0668 | 0.0645 | 0.4036 | 96.6 | 0.0669 | 0.0646 |
| (0.1, 0.1) |  | 0.0664 | 0 | 0.0097 | 0.0098 | 0.4011 | 27.8 | 0.0105 | 0.0582 | 0.4037 | 96.5 | 0.0688 | 0.0650 | 0.3990 | 96.5 | 0.0647 | 0.0584 | 0.4035 | 96.3 | 0.0672 | 0.0649 | 0.4040 | 96.5 | 0.0675 | 0.0651 |
| (0.3, 0.3) | 0 | -0.0004 | 95.0 | 0.0078 | 0.0077 | 0.0006 | 54.0 | 0.0079 | 0.0208 | 0.0001 | 95.0 | 0.0210 | 0.0208 | 0.0001 | 94.8 | 0.0209 | 0.0206 | 0.0001 | 94.8 | 0.0211 | 0.0208 | 0.0001 | 94.8 | 0.0211 | 0.0208 |
| (0.3, 0.1) |  | -0.0004 | 94.9 | 0.0078 | 0.0078 | 0.0006 | 55.0 | 0.0079 | 0.0210 | 0.0001 | 94.9 | 0.0212 | 0.0210 | 0.0001 | 95.2 | 0.0210 | 0.0208 | 0.0001 | 95.3 | 0.0212 | 0.0210 | 0.0001 | 95.0 | 0.0212 | 0.0210 |
| (0.1, 0.3) |  | -0.0003 | 94.4 | 0.0090 | 0.0090 | 0.0014 | 25.9 | 0.0091 | 0.0544 | 0.0010 | 95.5 | 0.0557 | 0.0548 | 0.0010 | 95.6 | 0.0537 | 0.0530 | 0.0010 | 95.5 | 0.0552 | 0.0548 | 0.0010 | 95.8 | 0.0553 | 0.0548 |
| (0.1, 0.1) |  | -0.0003 | 94.8 | 0.0090 | 0.0091 | 0.0013 | 26.5 | 0.0091 | 0.0553 | 0.0006 | 95.4 | 0.0564 | 0.0559 | 0.0006 | 94.6 | 0.0540 | 0.0539 | 0.0006 | 94.5 | 0.0557 | 0.0558 | 0.0090 | 94.4 | 0.0558 | 0.0559 |
| (0.3, 0.3) | -0.4 | -0.1504 | 0 | 0.0071 | 0.0073 | -0.3993 | 56.4 | 0.0079 | 0.0200 | -0.4004 | 94.0 | 0.0221 | 0.0222 | -0.3963 | 96.3 | 0.0219 | 0.0204 | -0.4004 | 94.7 | 0.0221 | 0.0222 | -0.4005 | 94.5 | 0.0221 | 0.0222 |
| (0.3, 0.1) |  | -0.1504 | 0 | 0.0071 | 0.0073 | -0.3993 | 55.8 | 0.0079 | 0.0199 | -0.4008 | 94.3 | 0.0223 | 0.0222 | -0.3964 | 96.2 | 0.0220 | 0.0202 | -0.4007 | 94.8 | 0.0223 | 0.0222 | -0.4008 | 94.6 | 0.0223 | 0.0222 |
| (0.1, 0.3) |  | -0.0671 | 0 | 0.0082 | 0.0084 | -0.3994 | 27.9 | 0.0091 | 0.0515 | -0.4018 | 95.7 | 0.0598 | 0.0595 | -0.3988 | 96.1 | 0.0568 | 0.0519 | -0.4018 | 94.9 | 0.0590 | 0.0594 | -0.4019 | 95.0 | 0.0591 | 0.0595 |
| (0.1, 0.1) |  | -0.0671 | 0 | 0.0082 | 0.0086 | -0.3994 | 28.2 | 0.0091 | 0.0518 | -0.4047 | 95.0 | 0.0606 | 0.0608 | -0.3974 | 95.8 | 0.0572 | 0.0526 | -0.4045 | 94.8 | 0.0595 | 0.0607 | -0.4049 | 94.7 | 0.0597 | 0.0609 |

Supplementary Table 2: Results from the simulation study of multivariable IVW (MVIVW), MV-LIML, CWBLS, MR-GRAPPLE, Debiased IVW and MRBEE to estimate the causal effect on disease progression, without causal relationship between exposure of interest and disease liability. $H_{GX}$ $H_{GD}$ $\beta_{XY}$Details as in Supplementary Table 1. Disease trait is simulated as a binary trait with prevalence 20%.

| Effect between $\boldsymbol{D}$ and $\boldsymbol{X}$ | Direction | MVIVW | | | | MV-LIML | | | | CWBLS | | | | MR-GRAPPLE | | | | Debiased IVW | | | | MRBEE | | | |
| --- | --- | --- | --- | --- | --- | --- | --- | --- | --- | --- | --- | --- | --- | --- | --- | --- | --- | --- | --- | --- | --- | --- | --- | --- | --- |
|  |  | EST | CVG | SE | SD | EST | CVG | SE | SD | EST | CVG | SE | SD | EST | CVG | SE | SD | EST | CVG | SE | SD | EST | CVG | SE | SD |
| 0.2 | $X$ to $D$ | 0.1339 | 0 | 0.0082 | 0.0084 | 0.4008 | 56.8 | 0.0090 | 0.0360 | 0.4007 | 94.7 | 0.0266 | 0.0265 | 0.3955 | 96.0 | 0.0263 | 0.0243 | 0.4007 | 95.1 | 0.0266 | 0.0265 | 0.4008 | 94.9 | 0.0266 | 0.0265 |
| 0.5 |  | 0.0863 | 0 | 0.0082 | 0.0081 | 0.4007 | 50.3 | 0.0092 | 0.0281 | 0.4021 | 95.1 | 0.0334 | 0.0335 | 0.3975 | 96.2 | 0.0326 | 0.0291 | 0.4021 | 95.5 | 0.0334 | 0.0335 | 0.4023 | 95.3 | 0.0334 | 0.0335 |
| -0.2 |  | 0.1527 | 0 | 0.0085 | 0.0086 | 0.4011 | 55.8 | 0.0090 | 0.0246 | 0.4000 | 94.6 | 0.0261 | 0.0259 | 0.3957 | 95.7 | 0.0259 | 0.0242 | 0.4000 | 95.0 | 0.0262 | 0.0259 | 0.4001 | 95.0 | 0.0262 | 0.0259 |
| -0.5 |  | 0.1312 | 0 | 0.0086 | 0.0087 | 0.4014 | 48.0 | 0.0092 | 0.0287 | 0.4005 | 95.0 | 0.0321 | 0.0320 | 0.3998 | 95.4 | 0.0315 | 0.0292 | 0.4005 | 94.8 | 0.0320 | 0.0320 | 0.4006 | 94.8 | 0.0320 | 0.0320 |
| 0.2 | $D$ to $X$ | 0.1394 | 0 | 0.0083 | 0.0082 | 0.4003 | 56.1 | 0.0091 | 0.0285 | 0.4004 | 95.6 | 0.0262 | 0.0248 | 0.3977 | 96.8 | 0.0257 | 0.0228 | 0.4004 | 95.5 | 0.0260 | 0.0224 | 0.3962 | 96.6 | 0.0260 | 0.0248 |
| 0.5 |  | 0.1196 | 0 | 0.0080 | 0.0078 | 0.4005 | 52.4 | 0.0089 | 0.0305 | 0.4009 | 95.9 | 0.0289 | 0.0271 | 0.3987 | 96.9 | 0.0283 | 0.0243 | 0.4009 | 95.7 | 0.0288 | 0.0271 | 0.401 | 95.7 | 0.0288 | 0.0270 |
| -0.2 |  | 0.1581 | 0 | 0.0083 | 0.0084 | 0.4002 | 55.1 | 0.0089 | 0.0191 | 0.4001 | 94.6 | 0.0253 | 0.0248 | 0.3991 | 95.7 | 0.0248 | 0.0232 | 0.4001 | 95.3 | 0.0251 | 0.0248 | 0.4002 | 95.3 | 0.0251 | 0.0248 |
| -0.5 |  | 0.1641 | 0 | 0.0080 | 0.0082 | 0.4003 | 50.2 | 0.0084 | 0.0451 | 0.4002 | 94.7 | 0.0264 | 0.0265 | 0.3995 | 95.2 | 0.0259 | 0.0248 | 0.4002 | 94.4 | 0.0262 | 0.0265 | 0.4002 | 94.6 | 0.0263 | 0.0265 |

Supplementary Table 3: Results from the simulation study of multivariable IVW (MVIVW), MV-LIML, CWBLS, MR-GRAPPLE, Debiased IVW and MRBEE to estimate the causal effect on disease progression $\beta_{XY}=0.4$, with causal relationship between exposure of interest and disease liability under different magnitudes and directions. Heritabilities of the exposure and disease trait$H_{GX}$ $H_{GD}$ are both fixed at 0.3. EST, mean estimate over 1000 simulations; CVG, coverage of 95% confidence interval; SE, mean of estimated standard errors; SD, empirical standard deviation of estimates. Disease trait is simulated as a continuous trait.

| $\left( h_{GX}^{2},h_{GD}^{2},h_{GY}^{2} \right)H_{GX}$ $H_{GD}$ $H_{GY}$ | $\boldsymbol{\beta}_{\boldsymbol{XY}}$ | MVIVW | | | | MV-LIML | | | | CWBLS | | | | MR-GRAPPLE | | | | Debiased IVW | | | | MRBEE | | | |
| --- | --- | --- | --- | --- | --- | --- | --- | --- | --- | --- | --- | --- | --- | --- | --- | --- | --- | --- | --- | --- | --- | --- | --- | --- | --- |
|  |  | EST | CVG | SE | SD | EST | CVG | SE | SD | EST | CVG | SE | SD | EST | CVG | SE | SD | EST | CVG | SE | SD | EST | CVG | SE | SD |
| (0.3, 0.3, 0.3) | 0.4 | 0.1495 | 0 | 0.0084 | 0.0102 | 0.8853 | 0 | 0.0115 | 0.0259 | 0.4003 | 95.4 | 0.0301 | 0.0290 | 0.4116 | 93.1 | 0.0299 | 0.0305 | 0.4003 | 95.9 | 0.0301 | 0.0290 | 0.4004 | 95.7 | 0.0301 | 0.0290 |
|  | 0 | -0.0004 | 89.8 | 0.0078 | 0.0096 | -0.0058 | 17.0 | 0.0111 | 0.1237 | -0.0007 | 94.7 | 0.0268 | 0.0261 | -0.0018 | 95.1 | 0.0285 | 0.0280 | -0.0007 | 95.6 | 0.0268 | 0.0261 | -0.0007 | 95.6 | 0.0268 | 0.0261 |
|  | -0.4 | -0.1503 | 0 | 0.0071 | 0.0073 | -0.8948 | 0 | 0.0079 | 0.0200 | -0.4004 | 94.0 | 0.0221 | 0.0222 | -0.3963 | 96.3 | 0.0219 | 0.0204 | -0.4004 | 94.7 | 0.0221 | 0.0222 | -0.4005 | 94.5 | 0.0221 | 0.0222 |
| (0.3, 0.3, 0.1) | 0.4 | 0.1496 | 0 | 0.0084 | 0.0092 | 0.4945 | 0 | 0.0095 | 0.0194 | 0.4007 | 94.7 | 0.0269 | 0.0268 | 0.4020 | 95.2 | 0.0261 | 0.0276 | 0.4007 | 95.0 | 0.0269 | 0.0268 | 0.4008 | 95.1 | 0.0269 | 0.0268 |
|  | 0 | -0.0003 | 93.4 | 0.0078 | 0.0086 | -0.0010 | 48.6 | 0.0080 | 0.0273 | -0.0004 | 94.3 | 0.0232 | 0.0234 | -0.0006 | 94.4 | 0.0234 | 0.0237 | -0.0004 | 94.6 | 0.0232 | 0.0234 | -0.0004 | 94.6 | 0.0232 | 0.0235 |
|  | -0.4 | -0.1502 | 0 | 0.0071 | 0.0080 | -0.5041 | 0 | 0.0084 | 0.0137 | -0.4013 | 94.9 | 0.0242 | 0.0241 | -0.4048 | 93.0 | 0.0230 | 0.0245 | -0.4013 | 95.2 | 0.0241 | 0.0241 | -0.4014 | 95.0 | 0.0242 | 0.0241 |
| (0.3, 0.1, 0.3) | 0.4 | 0.1495 | 0 | 0.0084 | 0.0101 | 0.9784 | 0 | 0.0115 | 0.0541 | 0.4005 | 95.5 | 0.0302 | 0.0291 | 0.4116 | 93.4 | 0.0301 | 0.0306 | 0.4005 | 95.9 | 0.0302 | 0.0291 | 0.4006 | 95.7 | 0.0302 | 0.0291 |
|  | 0 | -0.0004 | 90.4 | 0.0078 | 0.0096 | -0.0283 | 6.1 | 0.0367 | 0.7481 | -0.0008 | 95.1 | 0.0270 | 0.0261 | -0.0018 | 95.0 | 0.0288 | 0.0282 | -0.0008 | 95.8 | 0.0269 | 0.0261 | -0.0008 | 95.8 | 0.0269 | 0.0261 |
|  | -0.4 | -0.1503 | 0 | 0.0071 | 0.0088 | -0.9168 | 3.3 | 0.0112 | 0.1883 | -0.4021 | 96.5 | 0.0278 | 0.0264 | -0.4225 | 87.5 | 0.0277 | 0.0277 | -0.4021 | 96.6 | 0.0277 | 0.0264 | -0.4022 | 96.6 | 0.0278 | 0.0264 |
| (0.3, 0.1, 0.1) | 0.4 | 0.1496 | 0 | 0.0084 | 0.0092 | 0.1770 | 0.2 | 0.0102 | 0.0856 | 0.4009 | 94.8 | 0.0271 | 0.0269 | 0.4021 | 93.3 | 0.0262 | 0.0278 | 0.4009 | 95.1 | 0.0270 | 0.0269 | 0.4010 | 95.1 | 0.0271 | 0.0269 |
|  | 0 | -0.0003 | 93.5 | 0.0077 | 0.0085 | -0.0040 | 7.7 | 0.0100 | 0.1378 | -0.0002 | 94.7 | 0.0233 | 0.0234 | -0.0004 | 95.0 | 0.0235 | 0.0237 | -0.0002 | 95.1 | 0.0232 | 0.0234 | -0.0002 | 95.0 | 0.0233 | 0.0234 |
|  | -0.4 | -0.1502 | 0 | 0.0071 | 0.0078 | -0.1987 | 0 | 0.0094 | 0.0651 | -0.4008 | 95.7 | 0.0242 | 0.0236 | -0.4045 | 93.6 | 0.0231 | 0.0241 | -0.4008 | 96.0 | 0.0242 | 0.0236 | -0.4009 | 96.0 | 0.0242 | 0.0237 |
| (0.1, 0.3, 0.3) | 0.4 | 0.0663 | 0 | 0.0097 | 0.0122 | 0.9651 | 5.2 | 0.0160 | 0.7766 | 0.4030 | 94.8 | 0.0807 | 0.0802 | 0.4396 | 90.3 | 0.0808 | 0.0872 | 0.4030 | 94.1 | 0.0798 | 0.0802 | 0.4032 | 94.5 | 0.0800 | 0.0803 |
|  | 0 | -0.0003 | 88.2 | 0.0090 | 0.0115 | -0.0034 | 19.5 | 0.0126 | 0.2557 | -0.0020 | 94.4 | 0.0712 | 0.0713 | -0.0047 | 94.6 | 0.0859 | 0.0873 | -0.0020 | 94.5 | 0.0704 | 0.0713 | -0.0020 | 94.6 | 0.0705 | 0.0713 |
|  | -0.4 | -0.0669 | 0 | 0.0082 | 0.0110 | -3.5115 | 0 | 0.0336 | 0.3469 | -0.4066 | 95.9 | 0.0745 | 0.0748 | -0.4701 | 83.9 | 0.0748 | 0.0806 | -0.4066 | 95.3 | 0.0735 | 0.0748 | -0.4069 | 95.2 | 0.0737 | 0.0749 |
| (0.1, 0.3, 0.1) | 0.4 | 0.0664 | 0 | 0.0097 | 0.0107 | 0.3233 | 7.2 | 0.0105 | 0.1207 | 0.4037 | 94.9 | 0.0725 | 0.0722 | 0.4081 | 92.4 | 0.0680 | 0.0742 | 0.4037 | 94.8 | 0.0716 | 0.0722 | 0.4039 | 95.0 | 0.0718 | 0.0723 |
|  | 0 | -0.0002 | 93.0 | 0.009 | 0.0100 | -0.0006 | 47.5 | 0.0093 | 0.0306 | -0.0012 | 94.8 | 0.0617 | 0.0620 | -0.0017 | 94.4 | 0.0627 | 0.0638 | -0.0012 | 94.4 | 0.0609 | 0.0620 | -0.0012 | 94.6 | 0.0610 | 0.0621 |
|  | -0.4 | -0.0668 | 0 | 0.0082 | 0.0093 | -0.6452 | 5.6 | 0.0103 | 0.1477 | -0.4062 | 96.0 | 0.0652 | 0.0641 | -0.4157 | 92.6 | 0.0598 | 0.0651 | -0.4062 | 95.8 | 0.0642 | 0.0641 | -0.4064 | 95.8 | 0.0643 | 0.0642 |
| (0.1, 0.1, 0.3) | 0.4 | 0.0663 | 0 | 0.0097 | 0.0121 | 3.5203 | 0 | 0.0398 | 1.0431 | 0.4036 | 95.1 | 0.0814 | 0.0806 | 0.4398 | 90.3 | 0.0813 | 0.0878 | 0.4036 | 94.1 | 0.0802 | 0.0806 | 0.4039 | 94.7 | 0.0804 | 0.0807 |
|  | 0 | -0.0003 | 87.9 | 0.0090 | 0.0115 | 0.0795 | 3.0 | 0.0594 | 4.8149 | -0.0020 | 94.5 | 0.0719 | 0.0715 | -0.0046 | 94.2 | 0.0864 | 0.0875 | -0.0020 | 94.5 | 0.0707 | 0.0715 | -0.0020 | 94.7 | 0.0709 | 0.0715 |
|  | -0.4 | -0.0669 | 0 | 0.0082 | 0.0109 | -3.7003 | 0.4 | 0.0445 | 1.6024 | -0.4074 | 96.1 | 0.0753 | 0.0751 | -0.4693 | 84.2 | 0.0754 | 0.0811 | -0.4074 | 95.2 | 0.0739 | 0.0751 | -0.4076 | 95.1 | 0.0741 | 0.0752 |
| (0.1, 0.1, 0.1) | 0.4 | 0.0664 | 0 | 0.0097 | 0.0107 | 0.8640 | 0 | 0.0133 | 0.0962 | 0.4043 | 95.2 | 0.0732 | 0.0726 | 0.4087 | 92.2 | 0.0684 | 0.0748 | 0.4043 | 94.8 | 0.0720 | 0.0726 | 0.4046 | 95.0 | 0.0722 | 0.0727 |
|  | 0 | -0.0002 | 93.3 | 0.0089 | 0.0099 | -0.0107 | 7.9 | 0.0134 | 0.3369 | -0.0012 | 94.8 | 0.0622 | 0.0623 | -0.0018 | 94.5 | 0.0631 | 0.0641 | -0.0012 | 94.3 | 0.0612 | 0.0623 | -0.0012 | 94.4 | 0.0614 | 0.0623 |
|  | -0.4 | -0.0668 | 0 | 0.0082 | 0.0094 | -0.9745 | 0 | 0.0132 | 0.2307 | -0.4059 | 95.3 | 0.0657 | 0.0662 | -0.4161 | 90.8 | 0.0603 | 0.0681 | -0.4059 | 94.7 | 0.0646 | 0.0662 | -0.4062 | 94.4 | 0.0648 | 0.0663 |

Supplementary Table 4: Results from the simulation study of multivariable IVW (MVIVW), MV-LIML, CWBLS, MR-GRAPPLE, Debiased IVW and MRBEE with all invalid genetic instruments. $h_{GX}^{2}$ and $G$ $Y$ $H_{GX}$ $H_{GD}h_{GD}^{2}$ $H_{GY}$ are heritabilities of the exposure and disease trait. $h_{GY}^{2}$ is the variance of direct pleiotropic effects on disease progression. $\beta_{XY}$ is the true causal effect between the exposure and disease progression. EST, mean estimate over 1000 simulations; CVG, coverage of 95% confidence interval; SE, mean of estimated standard errors; SD, empirical standard deviation of estimates. Disease trait is simulated as a continuous trait.

# R package: *ColliderBias*

We have designed an R package to implement instrument effect regression in the main paper, built on the use of CWLS [2]. We name this package *ColliderBias* which consists of two main functions. The first function is called *methodCB*, which is the implementation of CWLS to adjust for collider bias and weak instrument bias when conditioning or stratifying on a variable that is associated with the outcome of interest [2]. The use of CWLS can be switched to other methods such as MR-RAPS and Slope-Hunter [3,4]. The second one is called *CWBLS* to apply CWBLS with generalised instrument effect regression. The package is open-source and can be found at <https://github.com/SiyangCai/ColliderBias>.

We did not include MV-LIML in the package as the original code can be found in the supplementary material of [5] (<https://pubmed.ncbi.nlm.nih.gov/35638254/>). We made a slight modification in the simulations of this paper. This is because the original code of MV-LIML is not computationally efficient against a large number of uncorrelated instruments. In our simulations we assume the matrix $\Omega\left( \theta\right)$ is diagonal, boosting efficiency by replacing matrix multiplications by scalar multiplications.

The package of MR-GRAPPLE[6] can be found at <https://github.com/jingshuw/GRAPPLE/tree/master>. The package of Debiased IVW [7] can be found at <https://github.com/tye27/mr.divw>. The package of MRBEE [8] can be found at <https://github.com/noahlorinczcomi/MRBEE>.

To illustrate the efficiency of the methods used in the main paper, we have run a sample dataset to record the run time according to the scenario described in Table 1. Supplementary Table 5 shows the run times. Note that using MV-LIML with the original code does not return any results due to excessive memory usage. It is clear that all other methods obtain the results almost instantly, except for MR-GRAPPLE which takes more than 2 minutes. However, the difference among the run time can be neglibible when using a smaller dataset, but more significant if a much larger dataset is used, e.g. a pruned dataset with high imputation level based on a large GWAS.

| Time to run (sec) | CWBLS | MV-LIML (original) | MV-LIML (simplified) | MR-GRAPPLE | Debiased IVW | MRBEE |
| --- | --- | --- | --- | --- | --- | --- |
|  | 0.6 | N/A | 0.2 | 149 | 19 | 0.5 |

Supplementary Table 5: Run time of methods in the main paper, using a sample dataset of 10,000 instruments generated in the scenario of Table 1.

# References

1. Dudbridge F, Allen RJ, Sheehan NA, Schmidt AF, Lee JC, Jenkins RG, et al. Adjustment for index event bias in genome-wide association studies of subsequent events. Nat Commun. 2019;10.

2. Cai S, Hartley A, Mahmoud O, Tilling K, Dudbridge F. Adjusting for collider bias in genetic association studies using instrumental variable methods. Genet Epidemiol. 2022;46:303–16.

3. Mahmoud O, Dudbridge F, Davey Smith G, Munafo M, Tilling K. A robust method for collider bias correction in conditional genome-wide association studies. Nat Commun. 2022;13.

4. Zhao Q, Wang J, Hemani G, Bowden J, Small DS. Statistical inference in two-sample summary-data Mendelian randomization using robust adjusted profile score. Ann Stat. 2020;48:1742–69.

5. Batool F, Patel A, Gill D, Burgess S. Disentangling the effects of traits with shared clustered genetic predictors using multivariable Mendelian randomization. Genet Epidemiol. 2022;46:415–29.

6. Wang J, Zhao Q, Bowden J, Hemani G, Smith GD, Small DS, et al. Causal inference for heritable phenotypic risk factors using heterogeneous genetic instruments. PLoS Genet. 2021;17.

7. Wu Y, Kang H, Ye T. Debiased Multivariable Mendelian Randomization. 2024; Available from: http://arxiv.org/abs/2402.00307

8. Lorincz-Comi N, Yang Y, Li G, Zhu X. MRBEE: A bias-corrected multivariable Mendelian randomization method. Human Genetics and Genomics Advances. 2024;5.
